# Template-based combinatorial enumeration of virtual compound libraries for lipids

**DOI:** 10.1186/1758-2946-4-23

**Published:** 2012-09-25

**Authors:** Manish Sud, Eoin Fahy, Shankar Subramaniam

**Affiliations:** 1 San Diego Supercomputer Center, University of California San Diego, 9500, Gilman Drive, La Jolla, CA 92032, USA; 2 Departments of Bioengineering, Chemistry and Biochemistry, University of California San Diego, 9500 Gilman Drive, La Jolla, CA 92093, USA

## Abstract

A variety of software packages are available for the combinatorial enumeration of virtual libraries for small molecules, starting from specifications of core scaffolds with attachments points and lists of R-groups as SMILES or SD files. Although SD files include atomic coordinates for core scaffolds and R-groups, it is not possible to control 2-dimensional (2D) layout of the enumerated structures generated for virtual compound libraries because different packages generate different 2D representations for the same structure. We have developed a software package called LipidMapsTools for the template-based combinatorial enumeration of virtual compound libraries for lipids. Virtual libraries are enumerated for the specified lipid abbreviations using matching lists of pre-defined templates and chain abbreviations, instead of core scaffolds and lists of R-groups provided by the user. 2D structures of the enumerated lipids are drawn in a specific and consistent fashion adhering to the framework for representing lipid structures proposed by the LIPID MAPS consortium. LipidMapsTools is lightweight, relatively fast and contains no external dependencies. It is an open source package and freely available under the terms of the modified BSD license.

## Background

The combinatorial virtual library enumeration methodology is routinely used during the early stages of the small molecule drug discovery cycle. Virtual compound libraries containing a large of number molecules are generated and ranked based on various calculated/predicted characteristics such as physicochemical properties, activity, specificity, solubility, etc. A set of top ranked compounds are selected and synthesized/acquired for further investigation using experimental techniques [1-7]. A variety of software packages are available for the combinatorial enumeration of virtual compound libraries. These tools fall into three broad categories: open source or freely available packages [8-12]; commercially available packages [13-21]; proprietary software packages implemented for internal use on top of custom or commercial software libraries [22-25]. Although implementation details might differ, all virtual library enumeration packages deploy similar general strategy to generate virtual compound libraries. A core scaffold along with attachment points for R-groups is specified and lists of R-groups are provided by the user. Options to incorporate linkers between the core scaffold and R-groups are also available in some packages. The core scaffold, R-groups and linkers are specified either as SMILES [26,27] or SD [28] files. All possible structures are enumerated by the combinatorial attachment of R-groups to the core scaffold along with the placement of any linkers between them and a virtual compound library is generated as a SMILES or SD file. The 2D structure representations generated for the compounds in virtual libraries are rather arbitrary. Although input SD files contain 2D atomic coordinate information for core scaffolds and R-groups, it is not possible to specify the exact orientation of R-groups around scaffolds for the structures enumerated for virtual libraries in any available software package, to the best of our knowledge. Different software packages end up generating completely different orientations of R-groups around scaffolds due to different internal strategies deployed for their optimal placement in the enumerated structures. Consequently, 2D structure layouts for the enumerated structures are not always consistent across software packages.

We have developed a software package called LipidMapsTools for the combinatorial enumeration of virtual compound libraries for lipids. Virtual libraries are enumerated for the specified lipid abbreviations using matching lists of pre-defined templates and chain abbreviations, instead of core scaffolds, linkers and lists of R-groups provided by the user as SMILES or SD files. 2D structures of the enumerated lipids are drawn in a specific fashion; their representation is consistent and adheres to the framework for representing lipid structures proposed by LIPID MAPS consortium [29,30]. The structure data for the enumerated virtual library is written to a SD file along with additional ontological information such as abbreviation, systematic name, category, main class, sub class, etc. LipidMapsTools is capable of generating large virtual compound libraries for lipids with minimal input from the user.

## Methodology

**Figure 1:**
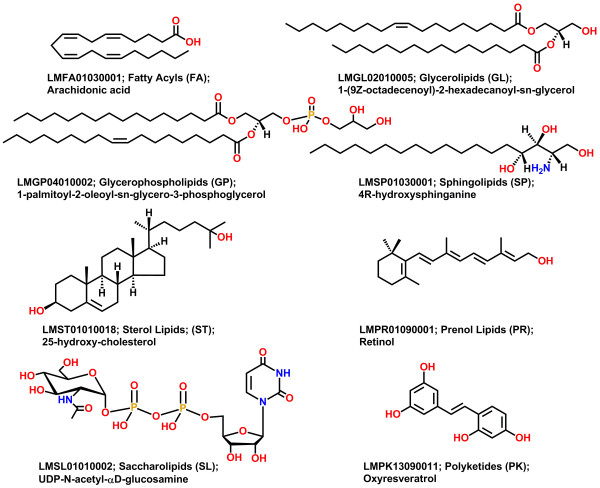
**Representative lipid structures from the LIPID MAPS structure database for different lipid categories. **LIPID MAPS LM ID, lipid category and name are shown under each structure.

We previously developed a LIPID MAPS Structure Database (LMSD) [31] containing structures and annotations of biologically relevant lipids. It is a relational database and currently contains over 37,000 structures. All lipids in the LMSD have been classified, named and drawn according to the comprehensive classification, nomenclature and drawing system [32,33] proposed by the LIPID MAPS consortium. Based on this classification system, lipids are divided into eight categories: fatty acyls (FA), glycerolipids (GL), glycerophospholipids (GP), sphingolipids (SP), sterol lipids (ST), prenol lipids (PR), saccharolipids (SL) and polyketides (PK). Each category is further divided into classes and subclasses. Figure 1 shows representative lipid structure for each lipid category along with its LIPID MAP ID (LM ID) and name.

**Figure 2:**
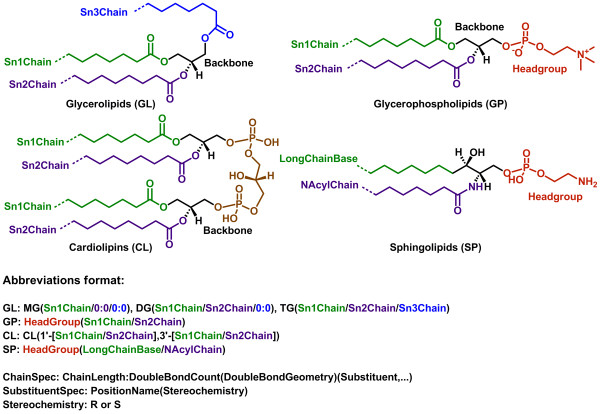
**Lipid categories amenable to the combinatorial enumeration of virtual compound libraries.** Lipid structure for each category is color coded to highlight chain, backbone and head group. Virtual libraries are generated from the specific lipid abbreviations by the combinatorial enumeration of all possible chains and head groups at various attachment points on the backbone. The lipid abbreviation format is shown below the structures. The format consists of the specification for chains along with the head group. The chain specification allows usage of * (asterisk) as a wild card character to indicate any chain length and number of double bonds along with their geometry during the generation of virtual libraries.

In general, the acid/acyl or head group is drawn on the right side and the hydrophobic radyl chain is shown on the left. For two out of the eight lipid categories, GL and GP, the radyl hydrocarbon chains - *sn1*, *sn3* and *sn3* chains at positions 1, 2 and 3 on the glycerol backbone corresponding to stereo chemical numbering (*sn*) scheme - are drawn with the chain termini on the left using attachment points on the template backbone and the appropriate head groups are shown on the right (Figure 2). The term radyl chain is used to represent acyl, alkyl or 1Z-alkenyl chains. For sphingolipids (SP), the two chains corresponding to a long chain base and an N-acyl chain are also drawn with the chain termini on the left of the ceramide template backbone and the head groups are on the right.

The three lipid categories of GL, GP and SP along with the cardiolipins (CL), a lipid class under GP, have a fixed backbone with chains and head groups attached to the specific attachment points on the backbone. These characteristics make these types of lipids amenable to the template-based combinatorial enumeration of virtual compound libraries, using the pre-defined lists of most likely chains and templates containing the appropriate head groups.

### Lipid abbreviations

The lipid abbreviation format (Figure 2) consists of the specifications for chains and head groups. The individual chain specifications are delimited by a backslash (/). For glycerolipids, three different lipid abbreviation formats are used: MG(*sn1*Chain/0:0/0:0), DG(*sn1*Chain/*sn2*Chain/0:0) and TG(*sn1*Chain/*sn2*Chain/*sn3*Chain). MG, DG and TG refer to monoradylglycerols, diradylglycerols and triradylgylcerols. Representative examples of the lipid abbreviation format for GL are: MG(16:0/0:0/0:0), DG(18:1(9Z)/16:0/0:0) and TG(16:0/16:0/18:1(11E)).These abbreviations correspond to 1-hexadecanoyl-*sn*-glycerol, 1-(9Z-octadecenoyl)-2-hexadecanoyl-*sn*-glycerol and 1,2-dihexadecanoyl-3-(11E-octadecenoyl)-*sn*-glycerol respectively.

The glycerophospholipid abbreviation format consists of the specifications for a head group and two *sn* chains: Headgroup(*sn1*Chain/*sn2*Chain). The cardiolipins abbreviation format is similar to the glycerophospholipids with additional specifications for extra set of *sn* chains: CL(1'-[*sn1*Chain/*sn2*Chain],3'-[*sn1*Chain/*sn2*Chain]). It has two sets of *sn1* and *sn2* chains at two different glycerol backbones attached to two phospho groups that are further connected to another glycerol backbone at 1' and 3' positions; no head group is specified. Representative examples of lipid abbreviations for GP are: PC(16:0/20:4(5Z,8Z,11Z,14Z)) and PE(16:0/18:1(9Z)). These abbreviations correspond to hexadecanoyl-2-(5Z,8Z,11Z,14Z-eicosatetraenoyl)-*sn*-glycero-3-phosphocholine and hexadecanoyl-2-(9Z-octadecenoyl)-*sn*-glycero-3-phosphoethanolamine. The cardiolipin abbreviation, CL(1'-[16:0/18:1(11Z)],3'-[16:0/18:1(11Z)]), corresponds to 1',3'-Bis-[1-hexadecanoyl-2-(11Z-octadecenoyl)-*sn*-glycero-3-phospho]-*sn*-glycerol.

The sphingolipid abbreviation format includes the specifications of a long chain base and an N-acyl chain on the ceramide backbone along with the specification of a head group: Headgroup(LongChainBase/NAcylChain). One of the three letters - d, t, or m - must precede the chain length specifier of the long chain base; the format of rest of the long chain base and N-acyl chain abbreviation is similar to the format of chain abbreviation for other lipid categories. The letters t and m are used to represent 4R-hydroxy and 3-keto groups at positions 4 and 3 respectively in the long chain base. Representative examples of the lipid abbreviations format for SP are: Cer(d18:1(4E)/14:0), Cer(t18:0/18:2(9Z,12Z)) and Cer(m14:0/16:1(9Z)). These abbreviations correspond to N-(tetradecanoyl)-sphing-4-enine, N-(9Z,12Z-octadecadienoyl)-4R-hydroxy-sphinganine and N-(9Z-hexadecenoyl)-3-keto-tetradecasphinganine.

The chain abbreviation format consists of the specifications for chain length, number of double bonds along with their geometry, and substituents. The chain length specification is mandatory; all other specifications are optional. The substituent specification includes its name, position in the chain and an optional value for stereochemistry (R or S). The stereochemistry for the substituents is determined using CIP (Cahn-Ingold-Prelog) [34-36] priority rules. For example, the acyl chain specification 16:0 corresponds to hexadecanoyl, and 20:4(7E,10E,13E,16E) implies 7E,10E,13E,16E-eicosatetraenoyl. A hydroxyl group at position 6 with R stereochemistry in 18:2(2E,4E) acyl chain corresponding to 2E,4E-octadecadienoyl is shown as 18:2(2E,4E)(6OH(R)). Table 1 shows representative examples of the *sn* chain specifications available during the combinatorial enumeration of virtual compound libraries for GL, GP and CL. Complete lists of the *sn* chain specifications for GL, GP and CL are shown in Additional file 1: Table S1; the long chain bases and N-acyl chain lists are shown in Additional file 1: Table S2 and Table S3; and Additional file 1: Table S4 provides the list of all available substituents.

**Table 1:** **Representative examples of chains available for *sn *positions during the combinatorial enumeration of virtual compound libraries**

**Abbreviation**	**Name**	**Abbreviation**	**Name**
2:0	acetyl	3:0	propionyl
8:0	octanoyl	9:0	nonanoyl
16:0	hexadecanoyl	16:0e	hexadecyl
18:0e	octadecyl	18:0p	octadecyl
18:0p	1Z-octadecenyl	P-18:0	1Z-octadecenyl
18:1(6Z)	6Z-octadecenoyl	18:1(7Z)	7Z-octadecenoyl
18:2e(1Z,9Z)	1Z,9Z-octadecadienyl	O-18:2(1Z,9Z)	1Z,9Z-octadecadienyl
20:0	eicosanoyl	20:0e	eicosyl
20:3(5Z,8Z,11Z)	5Z,8Z,11Z-eicosatrienoyl	20:4(5Z,8Z,11Z,13E)	5Z,8Z,11Z,13E-eicosatetraenoyl
20:4(7E,10E,13E,16E)	7E,10E,13E,16E-eicosatetraenoyl	20:5(5Z,8Z,11Z,14Z,17Z)	5Z,8Z,11Z,14Z,17Z-eicosapentaenoyl
21:0	heneicosanoyl	22:0	docosenyl
23:0	tricosanoyl	24:0	tetracosanoyl
24:1(15Z)	15Z-tetracosenoyl	24:4(5Z,8Z,11Z,14Z)	5Z,8Z,11Z,14Z-tetracosatetraenoyl
25:0	pentacosanoyl	26:0	hexacosanoyl
26:1(5Z)	5Z-hexacosenoyl	26:2(5Z,9Z)	5Z,9Z-hexacosadienoyl
35:0	pentatriacontanoyl	36:0	hexatriacontanoyl
37:0	heptatriacontanoyl	39:0	nonatriacontanoyl

Additional file 1: Table S1 shows complete list of chain abbreviations available during the virtual library enumeration for the lipid abbreviations containing wild cards.

Wild card specifications of chain lengths and double bonds along with their geometry are supported in the lipid abbreviation format, in order to specify a set of lipids to enumerate from the pre-defined lists of most likely *sn* chain abbreviations and head groups. Allowed wild card characters are: * (asterisk), + (plus), - (minus), > (greater than) and < (less than). The wild card character * is used for the specification of chain length and number of double bonds along with their geometry. It refers to all available chain abbreviations for lipids. The wild card characters + and - refer to even and odd chain lengths. The wild card characters > and < are used along with a number after them to indicate chain lengths greater than or less than a specified number; these are only valid for chain length specifications. For example, the lipid abbreviation TG(*/*/*) or TG(*:*/*:*/*:*) represents all possible triradylglycerolipid structures. The abbreviation DG(*:2/*:1(9Z)/0:0) corresponds to all possible diradylglycerolipid structures containing 2 double bonds in *sn1* chains and a specific double bond in *sn2* chains. The abbreviation PC(*- > 10 < 20:*/*+ > 16 < 24:*) implies all possible GP structures containing the phosphocholine head group, *sn1* chains with odd chain lengths greater than 10 and less than 20, and *sn2* chains with even chain lengths greater than 16 and less than 24. Table 2 shows representative examples of the lipid abbreviations for various lipid categories. Additional file 1: Tables S5, to S8 provide a comprehensive set of examples for the lipid abbreviations corresponding to GL, GP, CL and SP respectively.

**Table 2:** Representative examples of the commands for generating virtual compound libraries for different lipid categories

**Command**	**Description**
GLStrGen.pl “*(*:*/*:*/*:*)”	Enumerate all possible glycerolipid structures
GLStrGen.pl “*(*:2/*:*/*:*)”	Enumerate all possible glycerolipid structures containing 2 double bonds in sn1 chains
GLStrGen.pl “DG(*:2/*:1(9Z)/0:0)”	Enumerate all possible diglycerols structures containing 2 double bonds in sn1 chains and a specific double bond in sn2 chain
GPStrGen.pl “*(*:*(*)/*:*(*))”	Enumerate all possible glycerophospholipid structures
GPStrGen.pl “*(*- > 10 < 20:*/*+ > 16 < 24:*)”	Enumerate all possible glycerophospholipid structures containing sn1 chains with odd chain length > 10 and < 20, and sn2 chains with even chain length > 16 and < 24
GPStrGen.pl “PC(18:1(11E)/*:*)”	Enumerate all possible glycerophospholipid structures containing phosphocholine (PC) headgroup and a specific sn1 chain
CLStrGen.pl “*(1'-[*:*/*:*],3'-[*:*/*:*])”	Enumerate all possible cardiolipin structures
CLStrGen.pl “*(1'-[18:2(9Z,12Z)/*:*],3'-[*:*/*:*])”	Enumerate all possible cardiolipin structures containing a specific sn1 chain at 1' position
SPStrGen.pl “*(*:*/*:*)”	Enumerate all possible sphingolipid structures
SPStrGen.pl “*(*:*/0:0)”	Enumerate all possible sphingolipid structures without any N-acyl
SPStrGen.pl “*(*-:*/*:*)”	Enumerate all possible sphingolipid structures containing odd long chain base lengths
SPStrGen.pl “*(*+ > 18:*/0:0)”	Enumerate all possible sphingolipid structures containing long chain bases with even chain length > 18 and no N-acyl chain

Additional file 1: Tables S5 to S8 show a comprehensive list of examples for generating virtual libraries for different lipid categories.

### Combinatorial library enumeration workflow

**Figure 3:**
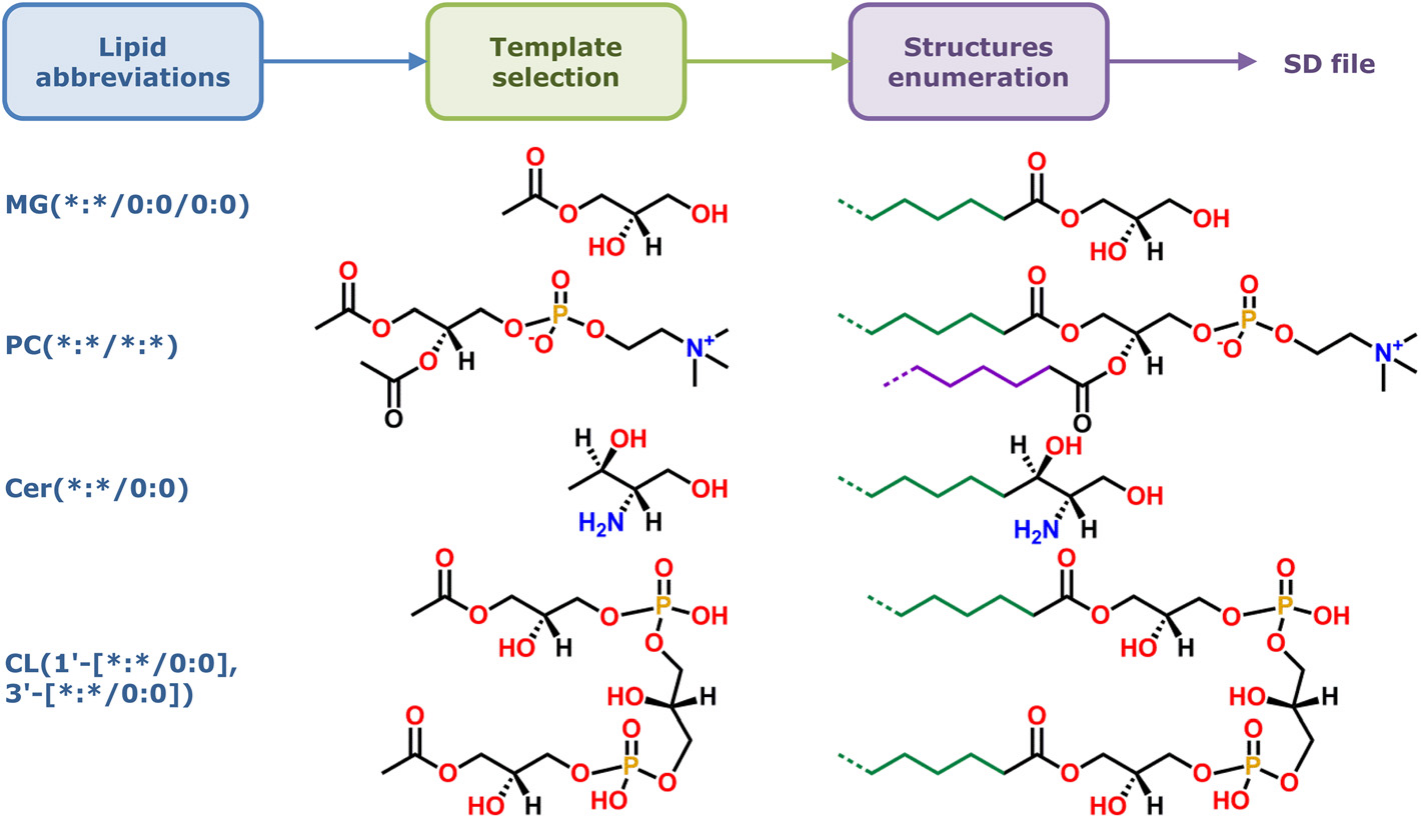
**Workflow for the template-based combinatorial enumeration of virtual compound libraries. **An appropriate lipid template structure is selected from a pre-defined list of templates for the specified lipid abbreviation. 2D structures of lipid templates are stored internally as MDL MOL strings and annotated with information regarding atom numbers and atomic coordinates for attachment points, number of existing carbon atoms in chains, head group name, etc. After an appropriate template has been identified and chains selected for the specified lipid abbreviation, a virtual compound library is generated by the combinatorial enumeration of all selected chains at appropriate attachment points on the template. A SD file is written out containing structure data along with abbreviation, systematic name, lipid category, main class, sub class, etc.

**Figure 4:**
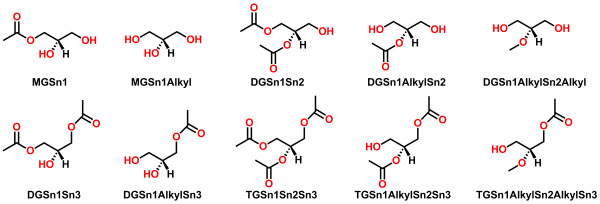
**Template structures for glycerolipids (GL). **Template IDs are shown below the structures. MG, DG and TG in template IDs correspond to monoradylglycerols, diradylglycerols and triradylglycerols.

**Figure 5:**
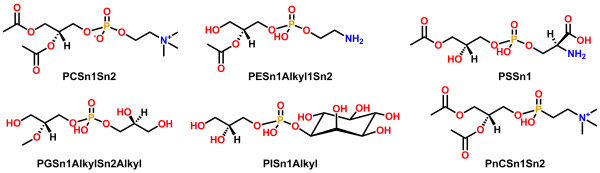
**Representative examples of template structures for glycerophospholipids (GP).** Additional file 1: Figure S1 shows complete list of supported template structures for GP. Template IDs containing head group specification are shown below the structures. The head group abbreviations shown in template IDs are: PC (Glycerophosphocholines), PE (Glycerophosphoethanolamines), PS (Glycerophosphoserines), PG (Glycerophosphoglycerols), PI (Glycerophosphoinositols), PnC (Glycerophosphonocholines).

**Figure 6:**
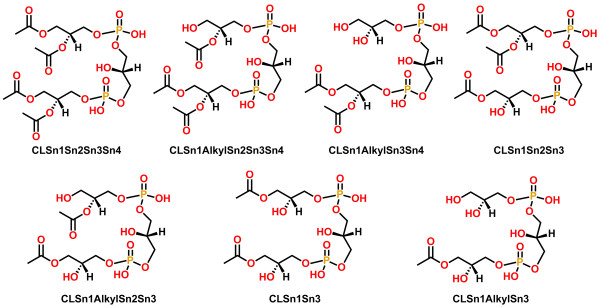
**Template structures for cardiolipins (CL). **Template IDs are shown below the structures.

**Figure 7:**
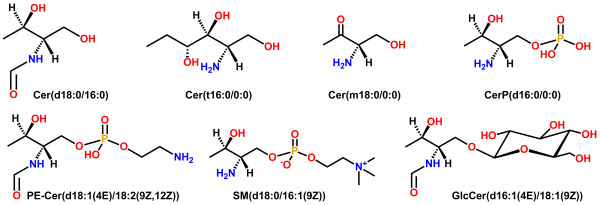
**Representative examples of template structures for sphingolipids (SP). **Additional file 1: Figure S2 shows complete list of supported template structures for SP. A representative example of an abbreviation matching a template is shown below the template structure. The head group abbreviations are: ceramide, 4R-hydroxy-ceramide, 3-keto-ceramide, CerP (ceramide-1-phosphate), PE-Cer (ceramide-1-phosphoethanolamine), SM (sphingomyelin), GlcCer (1-β-glucosyl-ceramide).

Figure 3 provides an overview of the methodology used by LipidMapsTools for the template-based combinatorial enumeration of virtual compound libraries for lipids. The process of virtual library enumeration starts out by the identification of a lipid template for the specified lipid abbreviation. The abbreviation is analyzed to identify the presence or absence of chains and head groups. The chain abbreviations are parsed to retrieve the specified chain length and number of double bonds along with their geometry. Any wild cards specified for chains and head groups are identified and marked for further combinatorial enumeration. An appropriate template or set of templates are selected from a pre-defined list of templates that match the specified lipid abbreviations. The templates are internally stored as MDLMOL strings containing structure data, along with mapping of each template ID to additional information such as atom numbers and atomic coordinates for attachment points, number of chain carbons in the template, head group name, lipid category, etc. The examples of available templates for GL, GP, CL and SP are shown in Figure 4, Figure 5 , Figure 6 and Figure 7 respectively (The Complete lists of templates GP and SP are available in Additional file 1: Figure S1 and Figure S2). After an appropriate template or set of templates have been identified for the specified lipid abbreviation, chain abbreviations that match the specified abbreviation are retrieved from the pre-defined list of most likely chain abbreviations spanning chain lengths from 2 to 39 for GL, GP and CL, and the most likely long chain bases and N-acyl groups for SP. Appropriate head groups are selected from the lists of supported head groups for GP and SP (Additional file 1:Table S9 and Additional file 1: Table S10). A virtual compound library for lipids is generated by the combinatorial enumeration of all selected chains at appropriate attachment points on the lipid templates. A SD file is written out containing structure data along with additional ontological data such as lipid abbreviation, systematic name, lipid category, main class, sub class, etc. Table 2 shows representative examples of the lipid abbreviations and commands for generating virtual compound libraries for various lipid categories.

In addition to the complete enumeration of all possible structures for various lipid categories using wild card characters for chain lengths, number of double bond along with their geometries and head groups, the LipidMapsTools software package allows the generation of subsets of these virtual libraries corresponding to specific chain lengths, number of double bonds with specific double bond geometry and head groups. For example, the lipid abbreviation "CL(1'-[* > 17 < 21:*/* > 17 < 21:*],3'-[* > 17 < 21:*/* > 17 < 21:*])" generates a subset of virtual compound library for the cardiolipins containing *sn* chain lengths between 17 and 21.

## Implementation

**Figure 8:**
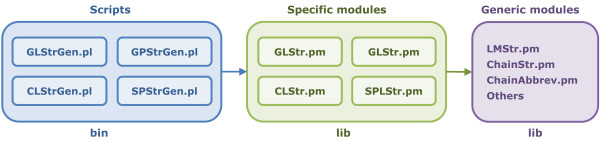
**Software architecture of the LipidMapsTools package. **The command line scripts make use of the specific module to generate virtual compound libraries, which in turn access the functionality from the generic modules to draw structures and generate additional ontological information such as abbreviation, systematic name, etc.

LipidMapsTools is implemented using the Perl [37,38] programming language and is available on a variety of platforms. The software architecture (Figure 8) consists of a *bin* sub-directory under the *lipidmapstools* root directory containing command line scripts for generating virtual compound libraries from the lipid abbreviations corresponding to the specific lipid categories, which in turn make use of the functionality available in Perl modules residing in the *lib* sub-directory to generate the structures along with the additional ontological information. The modules in the *lib* directory are divided into two categories: specific and generic modules. The specific modules contain functionality for virtual library generation corresponding to the specific lipid categories; the generic modules implement the core functionality such as chain abbreviation parsing, structure generation by attaching chains to the template, etc. The command line scripts make use of the specific modules to generate virtual compound libraries for lipids, which in turn rely on the core functionality to perform specific tasks. The separation of the core functionality from the lipid specific functionality facilitates the maintenance and enhancement of the LipidMapsTools package. Two external modules, TextUtil.pm and FileUtil.pm, available from an open source package MayaChemTools [39] are also distributed with LipidMapsTools. These modules provide functionality for processing data from text files and various file manipulation utilities.

The templates for lipid categories are stored in each category specific module as MDLMOL strings corresponding to template structure data, along with mapping of each template ID to additional information such as atom numbers and atomic coordinates for attachment points, number of chain carbons in the template, head group name, lipid category, main class, sub class, etc. No external MDLMOL data files are needed.

## Results and discussion

LipidMapTools provides command line scripts for the combinatorial enumeration of virtual compound libraries for lipids from the specified lipid abbreviations. Virtual compound libraries are generated by the combinatorial enumeration of most likely chains around the specific templates, with chain lengths varying from 2 to 39 containing specific number of double bonds and their geometry. Some radyl chains corresponding to alkyl and 1Z-alkenyl chains instead of acyl chains are skipped from *sn2* and *sn3* positions during the combinatorial enumeration, wherever they are not permitted by the LIPID MAPS classification scheme for lipids. For example, the LIPID MAPS classification scheme doesn’t contain any sub classes for alkyl and 1Z-alkenyl chains at *sn2* and *sn3* positions for the glycerolipids, and the structures corresponding to these chains at *sn2* and *sn3* positions are not generated.

Virtual compound libraries containing all possible structures for GL, GP, CL and SP are generated using the commands GLStrGen.pl "*(*/*/*)", GPStrGen.pl "*(*/*)", CLStrGen.pl "CL(1'-[*/*],3'-[*/*]) and SPStrGen.pl "*(*/*)" respectively. These command line scripts generate SD files containing the 2D structure data for all the enumerated structures along with additional information such as abbreviation, systematic name, chain length and double bond count, lipid category, main class, sub class, etc. The subsets of complete virtual libraries containing specific chain lengths and head groups are generated by their explicit specification in the specified lipid abbreviations. For example, the command GLStrGen.pl "PC(*:*/*:*)" generates a subset of GP virtual library containing all possible structures with the phosphocholine (PC) head group. A SP virtual library containing structures with the sphingnomyelin (SM) head group and the long chain bases between length 15 and 21 is generated by the following command: SPStrGen.pl "SM(* > 15 < 21:*/* > 15 < 21:*)". The complete lists of available head groups for GP and SP are shown in Additional file 1: Table S7 and Table S8.

The cardiolipins, a class of the glycerophospholipids, may contain up to 4 *sn* chains. Due to the combinatorial nature of the enumeration of all possible structures containing all 4 sn chains, the size of the CL virtual library gets to be quite large and it takes a substantial amount of time (Table 3) to generate the complete virtual library. A subset of the CL virtual library containing *sn* chain lengths between 17 and 21 can be generated using the following command: CLStrGen.pl "CL(1'-[* > 17 < 21:*/* > 17 < 21:*],3'-[* > 17 < 21:*/* > 17 < 21:*])". It is, however, feasible to speed up the task of the combinatorial enumeration of virtual compound libraries for CL and other lipid categories. Instead of running a single script to enumerate all possible structures corresponding to all combinations of chains, the virtual library generation script is started in parallel on multiple CPUs where each instance of the script enumerates a subset of a virtual compound library for a specific combinations of the chain lengths, and individual SD files are joined into a single SD file after all the jobs have completed.

**Table 3:** Command line format along with some relevant statistics for enumerating virtual compound libraries of lipids corresponding to various lipid categories

**Lipid category**	**Command**^**1**^	**Library size**^**2**^	**Speed**^**3**^	**Enumeration time**^**4**^
Glycerolipids (GL)	GLStrGen.pl “*(*/*/*)”	971,268	181.6	1 h 29 m 10s
Glycerophospholipids (SP)	GPStrGen.pl “*(*/*)”	211,615	213.2	16 m 33 s
Cardiolipins (CL)	CLStrGen.pl “*(1'-[*/*],3'-[*/*])”	88,584,132	129.3	190 h 18 m 36 s
	^5^CLStrGen.pl "CL(1'-[* > 17 < 21:*/* > 17 < 21:*],3'-[* > 17 < 21:*/* > 17 < 21:*])"	3,652,813	135.9	7 h 28 m 12 s
Sphingolipids (SP)	SPStrGen.pl” *(*/*)”	20,720	225.3	1 m 32 s

^1^Command line format for the combinatorial enumeration of virtual compound library of lipids for various lipid categories and generation of a SD file containing structures data along with additional ontological data such as abbreviation, systematic name, lipid category, main class, sub class, etc. ^2^Total number of lipid structures generated for virtual library in SD file. ^3^Average number of lipid structures generated per second. ^4^Time taken by the command line script to enumerate all possible lipid structures for each lipid category. Timing benchmarks were performed on a dual Intel^R ^Core^TM ^CPU 6700 (2.6 GHz) PC running Windows 7 Enterprise Service Pack 1 with 2.00 GB of RAM using single CPU per command line script. ^5^Cardiolipins command line format for generation of virtual compound library containing *sn *chains of lengths > 17 and < 21, representing a subset of the complete virtual library generated by CL command shown in preceding row.

The current focus of LipidMapsTools for the combinatorial enumeration of virtual compound libraries is on the mammalian lipids. The pre-defined lists of radyl chains and long chain bases contain the specifications for chain lengths with degrees of unsaturation that are most likely to occur in the mammalian lipids. Although these pre-defined lists are quite comprehensive, it is impossible to cover all the scenarios not only in terms of novel mammalian lipids but also different types of radyl chains or long chain bases that may be present in non-mammalian species such as plants, insects, bacteria, fungi and marine organisms. LipidMapsTools is designed to allow addition of new radyl chains and long chain bases in a relatively straight forward manner. The core Perl modules ChainAbbrev.pm and SPChainAbbev.pm contain the pre-defined lists of radyl chains and long chain bases respectively. After the pre-defined lists in the appropriate modules have been updated, the newly added radyl chains or long bases are available for the enumeration of virtual compound libraries through the command line scripts.

In addition to the combinatorial enumeration of virtual compound libraries for lipids from the specified abbreviations containing wild card characters, LipidMapsTools is capable of generating the specific lipid structures from the specific lipid abbreviations. For example, the command, GPStrGen.pl "PC(16:0/20:4(5Z,8Z,10E,14Z)(12OH[S]))", generates a SD file containing structure and ontological data for one glycerophospholipid corresponding to 1-hexadecanoyl-2-(12S-hydroxy-5Z,8Z,10E,14Z-eicosatetraenoyl)-*sn*-glycero-3-phosphocholine.

LipidMapsTools also provides the capability to generate individual lipid structures containing arbitrary specifications for radyl chains or long chain bases which are not present in the pre-defined lists available in the package, without requiring any customization by the user. This functionality is available in the scripts provided with the LipdMapsTools package through a command line option. For the lipid abbreviations containing arbitrary specifications, the structure generation methodology used in the LipidMapsTools package skips the step to confirm the presence of the specified radyl chains or long chain bases in the pre-defined lists of most likely chain lengths, and proceeds to generate the structure as long as the format of the specified abbreviation is valid. For example, the command, SPStrGen.pl --ChainAbbrevMode Arbitrary “SM(d30:4(4E,8E,12E,16E)/34:4(5Z,8Z,11Z,14Z)(16OH[R]))", parses the arbitrary specifications for the long chain base and N-acyl chain not present in the pre-defined lists available in the package, generates the appropriate sphingomyelin structure and writes it out to a SD file. This functionality facilitates the generation of individual structures for both mammalian and non-mammalian species containing radyl chains or long chain bases currently not present in the LipidMapsTools package. The capability to generate individual structures from the specific lipid abbreviation is quite useful for on-the-fly structure generation for populating databases and on line structure display.

## Conclusions

The LipidMapsTools software package has been developed for the template based combinatorial enumeration of virtual compound libraries for lipids. A set of command line scripts is provided to enumerate all possible structures corresponding to the specified lipid abbreviations without any additional input requirements from the user. It is relatively straight forward to generate subsets of complete virtual libraries by explicit specifications of chains and head groups in the lipid abbreviations. 2D structures of the enumerated lipids are drawn in a specific fashion; their representation is consistent and adheres to the framework for representing lipid structures proposed by LIPID MAPS consortium. The customization and enhancement of existing functionality along with development of new functionality is facilitated by modular nature of the software architecture. LipidMapsTools is under continuous development and we anticipate the addition of the new templates along with the radyl chains and long chain bases for both mammalian and non-mammalian lipid species in the future versions of the package.

## Availability and requirements

·**Project name:** lipidmapstools

·**Project home page:**http://www.lipidmaps.org/downloads/

·**Operating system(s):** Platform independent

·**Programming language:** Perl

·**Other requirements:** None

·**License:** Modified BSD License

·**Any restrictions to use by non-academics:** None

## Competing interests

MS also works as a part time independent consultant/contractor in the area of computational drug discovery and is involved in development of an open source software package called MayaChemTools. EF and SS declare that they have no competing interests.

## Authors’ contributions

MS performed research & development of the LipidMapsTools software package. EF contributed to design, prototype and testing of the package. SS provided feedback during the development cycle. MS drafted the manuscript and all co-authors contributed to the manuscript. All authors read and approved the final manuscript.

## Supplementary Material

Additional file 1 **Supplementary information as a PDF file. **Complete lists of the pre-defined chains specifications and examples of the enumeration of virtual compound libraries for GL, GP, CL and SP; lists of the head groups available for GP and SP; the structures of all available templates for GP and SP. Click here for file
