# Supplementary material for: Template-based combinatorial enumeration of virtual compound libraries for lipids
Source: J Cheminform. 2012 Sep 25;4:23. doi: 10.1186/1758-2946-4-23 (PMC3545849; doi:10.1186/1758-2946-4-23)
Supplement: Additional file 1 — Supplementary information as a PDF file. Complete lists of the pre-defined chains specifications and examples of the enumeration of virtual compound libraries for GL, GP, CL and SP; lists of the head groups available for GP and SP; the structures of all available templates for GP and SP. [file 1758-2946-4-23-S1.pdf]

Supplemental information

Template-based combinatorial enumeration of virtual compound libraries for lipids

Manish Sud[1*], Eoin Fahy[1*] and Shankar Subramaniam[1, 2*]

[1]San Diego Supercomputer Center, [2]Departments of Bioengineering, Chemistry and Biochemistry, University of California, San Diego, 9500 Gilman Drive, La Jolla, CA 92093

***Corresponding authors:** msud@san.rr.com, efahy@ucsd.edu, shankar@ucsd.edu

Table of contents

Table S1. Chains available for *sn1*, *sn2* and *sn3* positions during the combinatorial enumeration of virtual compound libraries for glycerolipids (GL), glycerophospholipids (GP) and cardiolipins (CL) abbreviations containing wild cards.

Table S2. Long chain bases available during the combinatorial enumeration of virtual compound libraries for sphingolipids (SP) abbreviations containing wild cards.

Table S3. N-acyl chains available during the combinatorial enumeration of virtual compound libraries for sphingolipids (SP) abbreviations containing wild cards.

Table S4. Substituents available during the generation of structures for the specific lipid abbreviations.

Table S5. Examples of lipid abbreviations for generation of glycerolipids (GL) structures using GLStrGen.pl script.

Table S6. Example of lipid abbreviations for generation of glycerophospholipids (GP) structures using GPStrGen.pl script.

Table S7. Example of lipid abbreviations for generation of cardiolipins (CL) structures using CLStrGen.pl script.

Table S8. Example of lipid abbreviations for generation of sphingolipids (SP) structures using SPStrGen.pl script.

Table S9. Head groups available during generation of structures for glycerophospholipids (GP).

Table S10. Head groups available during generation of structures for sphingolipids (SP).

Table S11. Chain name prefixes available for generation of systematic names.

Figure S1. Available template structures for glycerophospholipids (GP).

Figure S2. Available template structures for sphingolipids (SP).

Table S1. Chains available for *sn1*, *sn2* and *sn3* positions during the combinatorial enumeration of virtual compound libraries for glycerolipids (GL), glycerophospholipids (GP) and cardiolipins (CL) abbreviations containing wild cards.

**Abbreviation**	**Name**	**Abbreviation**	**Name**
2:0	acetyl	3:0	propionyl
4:0	butyryl	5:0	valeryl
6:0	hexanoyl	7:0	heptanoyl
8:0	octanoyl	9:0	nonanoyl
10:0	decanoyl	11:0	undecanoyl
12:0	dodecanoyl	13:0	tridecanoyl
14:0	tetradecanoyl	14:0e	tetradecyl
O-14:0	tetradecyl	14:1(9Z)	9Z-tetradecenoyl
15:0	pentadecanoyl	15:1(9Z)	9Z-pentadecenoyl
16:0	hexadecanoyl	16:0e	hexadecyl
O-16:0	hexadecyl	16:0p	1Z-hexadecenyl
P-16:0	1Z-hexadecenyl	O-16:1(1Z)	1Z-hexadecenyl
16:1e(9Z)	9Z-hexadecenyl	O-16:1(9Z)	9Z-hexadecenyl
16:1e(11Z)	11Z-hexadecenyl	O-16:1(11Z)	11Z-hexadecenyl
16:1(7Z)	7Z-hexadecenoyl	16:1(9Z)	9Z-hexadecenoyl
16:2e(1Z,9Z)	1Z,9Z-hexadecadienyl	O-16:2(1Z,9Z)	1Z,9Z-hexadecadienyl
16:2e(1Z,11Z)	1Z,11Z-hexadecadienyl	O-16:2(1Z,11Z)	1Z,11Z-hexadecadienyl
P-16:1(9Z)	1Z,9Z-hexadecadienyl	P-16:1(11Z)	1Z,11Z-hexadecadienyl
17:0	heptadecanoyl	17:1(9Z)	9Z-heptadecenoyl
17:2(9Z,12Z)	9Z,12Z-heptadecadienoyl	18:0	octadecanoyl
18:0e	octadecyl	O-18:0	octadecyl
18:0p	1Z-octadecenyl	P-18:0	1Z-octadecenyl
O-18:1(1Z)	1Z-octadecenyl	18:1e(9Z)	9Z-octadecenyl
O-18:1(9Z)	9Z-octadecenyl	18:1e(11Z)	11Z-octadecenyl
O-18:1(11Z)	11Z-octadecenyl	18:1(4E)	4E-octadecenoyl
18:1(6Z)	6Z-octadecenoyl	18:1(7Z)	7Z-octadecenoyl
18:1(9Z)	9Z-octadecenoyl	18:1(9E)	9E-octadecenoyl
18:1(11E)	11E-octadecenoyl	18:1(11Z)	11Z-octadecenoyl
18:1(13Z)	13Z-octadecenoyl	18:1(17Z)	13Z-octadecenoyl

18:2e(1Z,9Z)	1Z,9Z-octadecadienyl	O-18:2(1Z,9Z)	1Z,9Z-octadecadienyl
18:2e(1Z,11Z)	1Z,11Z-octadecadienyl	O-18:2(1Z,11Z)	1Z,11Z-octadecadienyl
P-18:1(9Z)	1Z,9Z-octadecadienyl	P-18:1(11Z)	1Z,11Z-octadecadienyl
18:2(2E,4E)	2E,4E-octadecadienoyl	18:2(6Z,9Z)	6Z,9Z-octadecadienoyl
18:2(9E,11E)	9E,11E-octadecadienoyl	18:2(9Z,11Z)	9Z,11Z-octadecadienoyl
18:2(9Z,12Z)	9Z,12Z-octadecadienoyl	18:2(9E,12E)	9E,12E-octadecadienoyl
18:3(6Z,9Z,12Z)	6Z,9Z,12Z-octadecatrienoyl	18:3(9Z,12Z,15Z)	9Z,12Z,15Z-octadecatrienoyl
18:4(6Z,9Z,12Z,15Z)	6Z,9Z,12Z,15Z-octadecatetraenoyl	18:4(9E,11E,13E,15E)	9E,11E,13E,15E-octadecatetraenoyl
19:0	nonadecanoyl	20:0	eicosanoyl
20:0e	eicosyl	O-20:0	eicosyl
20:0p	1Z-eicosenyl	P-20:0	1Z-eicosenyl
O-20:1(1Z)	1Z-eicosenyl	20:1e(9Z)	9Z-eicosenyl
O-20:1(9Z)	9Z-eicosenyl	20:1e(11Z)	11Z-eicosenyl
O-20:1(11Z)	11Z-eicosenyl	20:1(11Z)	11Z-eicosenoyl
20:1(11E)	11E-eicosenoyl	20:1(13Z)	13Z-eicosenoyl
20:1(13E)	13E-eicosenoyl	20:2e(1Z,9Z)	1Z,9Z-eicosadienyl
O-20:2(1Z,9Z)	1Z,9Z-eicosadienyl	20:2e(1Z,11Z)	1Z,11Z-eicosadienyl
O-20:2(1Z,11Z)	1Z,11Z-eicosadienyl	P-20:1(9Z)	1Z,9Z-eicosadienyl
P-20:1(11Z)	1Z,11Z-eicosadienyl	20:2(11Z,14Z)	11Z,14Z-eicosadienoyl
20:2(5Z,8Z)	5Z,8Z-eicosadienoyl	20:3(8Z,11Z,14Z)	8Z,11Z,14Z-eicosatrienoyl
20:3(5Z,8Z,11Z)	5Z,8Z,11Z-eicosatrienoyl	20:4(5Z,8Z,11Z,13E)	5Z,8Z,11Z,13E-eicosatetraenoyl
20:4(5Z,8Z,11Z,14Z)	5Z,8Z,11Z,14Z-eicosatetraenoyl	20:4(5Z,8Z,10E,14Z)	5Z,8Z,10E,14Z-eicosatetraenoyl
20:4(5E,8E,11E,14E)	5E,8E,11E,14E-eicosatetraenoyl	20:4(6E,8Z,11Z,14Z)	6E,8Z,11Z,14Z-eicosatetraenoyl
20:4(7E,10E,13E,16E)	7E,10E,13E,16E-eicosatetraenoyl	20:5(5Z,8Z,11Z,14Z,17Z)	5Z,8Z,11Z,14Z,17Z-eicosapentaenoyl
21:0	heneicosanoyl	22:0	docosenyl
22:0e	docosenyl	O-22:0	docosenyl
22:0p	1Z-docosenyl	P-22:0	1Z-docosenyl
O-22:1(1Z)	1Z-docosenyl	22:1e(9Z)	9Z-docosenyl
O-22:1(9Z)	9Z-docosenyl	22:1e(11Z)	11Z-docosenyl
O-22:1(11Z)	11Z-docosenyl	22:1(13Z)	13Z-docosenoyl

22:2e(1Z,9Z)	1Z,9Z-docosenyl	O-22:2(1Z,9Z)	1Z,9Z-docosenyl
22:2e(1Z,11Z)	1Z,11Z-docosenyl	O-22:2(1Z,11Z)	1Z,11Z-docosenyl
P-22:1(9Z)	1Z,9Z-docosenyl	P-22:1(11Z)	1Z,11Z-docosenyl
22:2(13Z,16Z)	13Z,16Z-docosadienoyl	22:4(7Z,10Z,13Z,16Z)	7Z,10Z,13Z,16Z-docosatetraenoyl
22:5(4Z,7Z,10Z,13Z,16Z)	4Z,7Z,10Z,13Z,16Z-docosapentaenoyl	22:5(7Z,10Z,13Z,16Z,19Z)	7Z,10Z,13Z,16Z,19Z-docosapentaenoyl
22:6(4Z,7Z,10Z,12E,16Z,19Z)	4Z,7Z,10Z,12E,16Z,19Z-docosahexaenoyl	22:6(4Z,7Z,10Z,13Z,16Z,19Z)	4Z,7Z,10Z,13Z,16Z,19Z-docosahexaenoyl
23:0	tricosanoyl	24:0	tetracosanoyl
24:1(15Z)	15Z-tetracosenoyl	24:4(5Z,8Z,11Z,14Z)	5Z,8Z,11Z,14Z-tetracosatetraenoyl
25:0	pentacosanoyl	26:0	hexacosanoyl
26:1(5Z)	5Z-hexacosenoyl	26:2(5Z,9Z)	5Z,9Z-hexacosadienoyl
26:2(5Z,9E)	5Z,9E-hexacosadienoyl	26:2(5E,9Z)	5Z,9E-hexacosadienoyl
27:0	heptacosanoyl	28:0	octacosanoyl
29:0	nonacosanoyl	30:0	triacontanoyl
31:0	hentriacontanoyl	32:0	dotriacontanoyl
33:0	triatricontanoyl	34:0	tetratriacontanoyl
35:0	pentatriacontanoyl	36:0	hexatriacontanoyl
37:0	heptatriacontanoyl	38:0	octatriacontanoyl
39:0	nonatriacontanoyl		

Table S2. Long chain bases available during the combinatorial enumeration of virtual compound libraries for sphingolipids (SP) abbreviations containing wild cards.

Abbreviation	Name	Abbreviation	Name
14:0	tetradecanoyl	14:1(4E)	4E-tetradecenoyl
14:1(8E)	8E-tetradecenoyl	14:2(4E,8E)	4E,8E-tetradecadienoyl
15:0	pentadecanoyl	15:1(4E)	4E-pentadecenoyl
15:1(8E)	8E-pentadecenoyl	15:2(4E,8E)	4E,8E-pentadecadienoyl
16:0	hexadecanoyl	16:1(4E)	4E-hexadecenoyl
16:1(8E)	8E-hexadecenoyl	16:1(4E,8E)	4E,8E-hexadecadienoyl
17:0	heptadecanoyl	17:1(4E)	4E-heptadecenoyl
17:1(8E)	8E-heptadecenoyl	17:1(4E,8E)	4E,8E-heptadecadienoyl
18:0	octadecanoyl	18:1(4E)	4E-octadecenoyl
18:1(8E)	8E-octadecenoyl	18:2(4E,8E)	4E,8E-octadecadienoyl
18:2(4E,14Z)	4E,14Z-octadecadienoyl	19:0	nonadecanoyl
19:1(4E)	4E-nonadecenoyl	19:1(8E)	8E-nonadecenoyl
19:1(4E,8E)	4E,8E-nonadecadienoyl	20:0	eicosanoyl
20:1(4E)	4E-eicosenoyl	20:1(8E)	8E-eicosenoyl
20:1(4E,8E)	4E,8E-eicosadienoyl	21:0	heneicosanoyl
21:1(4E)	4E-heneicosenoyl	21:1(8E)	8E-heneicosenoyl
21:1(4E,8E)	4E,8E-heneicosadienoyl	22:0	docosanoyl
22:1(4E)	4E-docosenoyl	22:1(8E)	8E-docosenoyl
22:1(4E,8E)	4E,8E-docosadienoyl		

Table S3. N-acyl chains available during the combinatorial enumeration of virtual compound libraries for sphingolipids (SP) abbreviations containing wild cards.

Abbreviation	Name	Abbreviation	Name
2:0	acetyl	10:0	decanoyl
12:0	dodecanoyl	13:0	tridecanoyl
14:0	tetradecanoyl	15:0	pentadecanoyl
16:0	hexadecanoyl	16:1(9Z)	9Z-hexadecenoyl
17:0	heptadecanoyl	17:1(9Z)	9Z-heptadecenoyl
18:0	octadecanoyl	18:1(9Z)	9Z-octadecenoyl
18:2(9Z,12Z)	9Z,12Z-octadecadienoyl	19:0	nonadecanoyl
20:0	eicosanoyl	20:1(11Z)	11Z-eicosenoyl
21:0	heneicosanoyl	22:0	docosanoyl
22:1(13Z)	13Z-docosenoyl	23:0	tricosanoyl
24:0	tetracosanoyl	24:1(15Z)	15Z-tetracosenoyl
24:4(5Z,8Z,11Z,14Z)	5Z,8Z,11Z,14Z-tetracosatetraenoyl	25:0	pentacosanoyl
26:0	hexacosanoyl	26:1(17Z)	17Z-hexacosenoyl
27:0	heptacosanoyl		

Table S4. Substituents available during the generation of structures for specific lipid abbreviations.

**Abbreviation**	**Name**	**Abbreviation**	**Name**	**Abbreviation**	**Name**
OH	hydroxy	NH2	amino	SH	thio
Me	methyl	Et	ethyl	Pr	propyl
OMe	methoxy	OAc	acetoxy	Ke	oxo
Ep	epoxy	Cp	cyclopropyl	My	methylene
OOH	hydroperoxy	Br	bromo	Cl	chloro
F	fluro	I	iodo	CN	cyno
NO2	nitro	COOH	carboxy	CHO	aldehyde

Table S5. Examples of lipid abbreviations for generation of glycerolipids (GL) structures using GLStrGen.pl script.

Abbreviation	Description
(/*/*)	Enumerate all possible structures
(:*/*:*/*:*)	Enumerate all possible structures
(:*(*)/*:*(*)/*:*(*))	Enumerate all possible structures
(:2/*:*/*:*)	Enumerate all possible structures containing 2 double bonds in *sn1* chains
(:2/*:1(9Z)/*:*)	Enumerate all possible structures containing 2 double bonds in *sn1* chains and one specific double bond in *sn2* chain
(+:*/*:*/*:*)	Enumerate all possible structures containing *sn1* chains with even chain lengths
(-:*/*:*/*:*)	Enumerate all possible structures containing *sn1* chains with odd chain lengths
(-:*/*+:*/*:*)	Enumerate all possible structures containing *sn1* chains with odd chain lengths and *sn2* chains with even lengths
(->12:*/*+>18:*/*:*)	Enumerate all possible structures containing *sn1* chains with odd chain lengths > 12 and *sn2* chains with even lengths > 18
(->12<20:*/*+>18<26:*/*:*)	Enumerate all possible structures containing *sn1* chains with odd chain lengths > 12 and < 20, and *sn2* chains with even lengths > 18 and < 26
MG(*:*/0:0/0:0)	Enumerate all possible monoradylglycerols structures
DG(*:*/*:*/0:0)	Enumerate all possible diradylglycerols structures
TG(*:*/*:*/*:*)	Enumerate all possible triadylglycerols structures
TG(18:1(11E)/*:*/*:*)	Enumerate all possible triadylglycerols structures containing a specific *sn1* chain
DG(*:2/*:1(9Z)/0:0)	Enumerate all possible diradylglycerols structures containing 2 double bonds in *sn1* chains and a specific double bond in *sn2* chain
TG(*->16:*/*+>12:*/*:*)	Enumerate all possible triadylglycerols structures containing *sn1* chains with odd chain length > 16 and *sn2* chains with even chain length > 12
MG(16:0/0:0/0:0)	Generate a specific monoacylglycerol structure
DG(18:1(11E)/16:0/0:0)	Generate a specific diradylglycerol structure
DG(18:1(11E)/16:0/0:0)[S]	Generate a specific diradylglycerol structure
TG(16:0/16:0/18:1(9Z))	Generate a specific triadylglycerol structure
DG(18:1(11E)/16:0/0:0)[iso2]	Enumerate two isomeric structures for diradylglycerols
TG(16:0/16:0/18:1(9Z))[iso3]	Enumerate three isomeric structures for triadylglycerols
TG(16:0/17:0/18:1(9Z))[iso6]	Enumerate six isomeric structures for triadylglycerols
TG(16:0(6Cl,8OH)/16:0/18:1(9Z))	Generate a specific triadylglycerol structure with substituents in *sn1* chain

Table S6. Example of lipid abbreviations for generation of glycerophospholipids (GP) structures using GPStrGen.pl script.

**Abbreviation**	**Description**
(/*)	Enumerate all possible structures
(:*/*:*)	Enumerate all possible structures
(:*(*)/*:*(*))	Enumerate all possible structures
(:*/0:0)	Enumerate all possible structures without any *sn2* chain
(:2/*:*)	Enumerate all possible structures containing 2 double bonds in *sn1* chains
(:2/*:1(9Z))	Enumerate all possible structures containing 2 double bonds in *sn1* chains and one specific double bond in *sn2* chain
(+:*/*:*)	Enumerate all possible structures containing *sn1* chain with even lengths
(-:*/*:*)	Enumerate all possible structures containing *sn1* chain with odd chain lengths
(+>10:*/0:0)	Enumerate all possible structures containing *sn1* chains with odd chain length > 10 and no *sn2* chains
(->10:*/*+>16:*)	Enumerate all possible structures containing *sn1* chains with odd chain length > 10 and *sn2* chains with even chain length > 16
(->10<20:*/*+>16<30:*)	Enumerate all possible structures containing *sn1* chains with odd chain length > 10 and < 20, and *sn2* chains with even chain length > 16 and < 30
PC(*:*/*:*)	Enumerate all possible structures containing phosphocholine (PC) headgroup
PC(18:1(11E)/*:*)	Enumerate all possible structures containing phosphocholine (PC) headgroup and a specific *sn1* chain
PC(16:0/0:0)	Generate a specific structure containing phosphocholine (PC) headgroup
PE(18:1(11E)/16:0)	Generate a specific structure containing phosphoethanolamine (PE) headgroup
PE(18:1(11E)/16:0)[S]	Generate a specific structure containing phosphoethanolamine (PE) headgroup with S stereochemistry on *sn2* backbone carbon
PC(16:0/0:0)[U]	Generate a specific structure containing phosphocholine (PC) headgroup with unknown stereochemistry on *sn2* backbone carbon
PS(18:1(11E)(6OH)/16:0(8Cl))	Generate a specific structure with phosphoserine (PS) headgroup with substituents in *sn1* and *sn2* chains

Table S7. Example of lipid abbreviations for generation of cardiolipins (CL) structures using CLStrGen.pl script.

**Abbreviation**	**Description**
CL(1'-[*/*],3'-[*/*])	Enumerate all possible structures
CL(1'-[*:*/*:*],3'-[*:*/*:*])	Enumerate all possible structures
CL(1'-[*:*(*)/*:*(*)],3'-[*:*(*)/*:*(*)])	Enumerate all possible structures
CL(1'-[*:2/*:*],3'-[*:*/*:*])	Enumerate all possible structures containing 2 double bonds in *sn1* chains at 1' position
CL(1'-[*:2/*:*1(9Z)],3'-[*:*/*:*])	Enumerate all possible structures containing 2 double bonds in *sn1* chains and one specific double bond in *sn2* chains at 1' position
CL(1'-[*+:*/*:*],3'-[*:*/*:*])	Enumerate all possible structures containing *sn1* chains with even chain lengths at 1' position
CL(1'-[*:*/*:*],3'-[*:*-/*:*])	Enumerate all possible structures containing *sn1* chains with odd chain lengths at 3' position
CL(1'-[*:*/*:*+],3'-[*:*/*:*-])	Enumerate all possible structures containing *sn2* chains with even chain lengths at 1' position and *sn2* chains with odd chain lengths at 3' position
CL(1'-[18:2(9Z,12Z)/*:*],3'-[*:*/*:*])	Enumerate all possible structures containing a specific *sn1* chain at 1' position
CL(1'-[*->12:*/+>18:*],3'-[*->14:*/+>20:*])	Enumerate all possible structures containing *sn1* chains with odd chain lengths > 12 and *sn2* chains with even chain lengths > 18 at 1' position, and *sn1* chains with odd chain lengths > 14 and *sn2* chains with even chain length > 20 at 3' position
CL(1'-[*>10<22:*/*>10<22:*],3'-[>10<22:*/>10<22:*])	Enumerate all possible structures containing *sn1* chains with chain lengths > 10 and *sn2* chains with chain lengths < 22 at 1' and 3' positions
CL(1'-[18:2(9Z,12Z)/18:2(9Z,12Z)],3'-[18:2(9Z,12Z)/18:2(9Z,12Z)])	Generate a specific structure
CL(1'-[18:2(9Z,12Z)/0:0],3'-[18:2(9Z,12Z)/0:0])	Generate a specific structure
CL(1'-[18:2(9Z,12Z)(5OH,14OH))/0:0],3'-[18:2(9Z,12Z)(6OH)/0:0])	Generate a specific structure with substituents

Table S8. Example of lipid abbreviations for generation of sphingolipids (SP) structures using SPStrGen.pl script.

Abbreviation	Description
(/*)	Enumerate all possible structures
(:*/*:*)	Enumerate all possible structures
(:*(*)/*:*(*))	Enumerate all possible structures
(:*/0:0)	Enumerate all possible structures with no N-acyl chains
(:2/*:*)	Enumerate all possible structures containing 2 double bonds in long chain bases
(:2/*:1(9Z))	Enumerate all possible structures containing 2 double bonds in long chain bases and one double bond in N-acyl chains
(+:*/*:*)	Enumerate all possible structures containing long chain base with even chain lengths
(-:*/*:*)	Enumerate all possible structures containing long chain base with odd chain lengths
(+>18:*/0:0)	Enumerate all possible structures containing long chain bases with even chain length > 18 and no N-acyl chain
(->18:*/*+>20:*)	Enumerate all possible structures containing long chain bases with odd length > 18 and N-acyl chains with even chain length > 20
(->10<20:*/*+>12<24:*)	Enumerate all possible structures containing long chain bases with odd length > 10 and < 20, and N-acyl chains with even chain length > 12 and < 24
Cer(d18:0/*:*)	Enumerate all possible sphinganine structures containing long chain base length 18
Cer(d19:1(4E)/*:*)	Enumerate all possible sphing-4-enine structures containing long chain base length 18
Cer(t18:0/*:*)	Enumerate all possible 4R-hydroxy-sphinganine structures containing long chain base length 18
Cer(d18:0/0:0)	Generate a specific sphinganine structure
Cer(d19:1(4E)/24:4(5Z,8Z,11Z,14Z))	Generate a specific sphing-4-enine structure
SM(d18:0/16:0)	Generate a specific sphinganine-1-phosphocholine structure
SM(d18:0/16:0(8OH))	Generate a specific sphinganine-1-phosphocholine structure with a substituent on N-acyl chain

Table S9. Head groups available during generation of structures for glycerophospholipids (GP).

Abbreviation	Name
PA	glycero-3-phosphate
PC	glycero-3-phosphocholine
PE	glycero-3-phosphoethanolamine
PENMe	glycero-3-phospho-N-methylethanolamine
PENMe2	glycero-3-phospho-N,N-dimethylethanolamine
PG	glycero-3-phospho-(1'-sn-glycerol)
PGP	glycero-3-phospho-(1'-sn-glycerol-3'-phosphate)
PI	glycero-3-phospho-(1'-myo-inositol)
PIP	glycero-3-phospho-(1'-myo-inositol-3'-phosphate)
PIP2[3',4']	glycero-3-phospho-(1'-myo-inositol-3',4'-biphosphate)
PIP2[3',5']	glycero-3-phospho-(1'-myo-inositol-3',5'-biphosphate)
PIP2[4',5']	glycero-3-phospho-(1'-myo-inositol-4',5'-biphosphate)
PIP3[3',4',5']	glycero-3-phospho-(1'-myo-inositol-3',4',5'-triphosphate)
PIP[3']	glycero-3-phospho-(1'-myo-inositol-3'-phosphate)
PIP[4']	glycero-3-phospho-(1'-myo-inositol-4'-phosphate)
PIP[5']	glycero-3-phospho-(1'-myo-inositol-5'-phosphate)
PPA	glycero-3-pyrophosphate
PS	glycero-3-phosphoserine
PnC	glycero-3-phosphonocholine
PnE	glycero-3-phosphonoethanolamine

Table S10. Head groups available during generation of structures for sphingolipids (SP).

Abbreviation	Headgroup name	Abbreviation name
CerP	1-phosphate	
PE-Cer	1-phosphoethanolamine	
PI-Cer	1-phospho-(1'-myo-inositol)	
GB3Cer	1-β-GB3	Galα1-4Galβ1-4Glcβ-Cer
GM3Cer	1-β-GM3	NeuAcα1-3Galβ1-4Glcβ-Cer
GM4Cer	1-β-GM4	NeuAcα1-3Galβ-Cer
GalCer	1-β-galactosyl	
GlcCer	1-β-glucosyl	
LacCer	1-β-lactosyl	Galβ1-4Glcβ-Cer
Lc3Cer	1-β-Lc3	GlcNAcβ1-4Galβ1-4Glcβ-Cer
Manb1-4GlcCer	1-β-Manb4Glc	Manβ1-4Glcβ-Cer
MolluCer	1-β-Mollu	Manβ1-3Manβ1-4Glcβ-Cer
SM	1-phosphocholine	
asialo-GM2Cer	1-β-asialo-GM2	GalNAcβ1-4Galβ1-4Glcβ-Cer
ArthroCer	1-β-Arthro	GlcNAcβ1-4Manβ1-4Glc-Cer
iGB3Cer	1-β-iGB3	Galα1-3Galβ1-4Glcβ-Cer

Table S11. Chain name prefixes available for generation of systematic names.

Length	Prefix	Length	Prefix	Length	Prefix
2	eth	3	prop	4	but
5	pent	6	hex	7	hept
8	oct	9	non	10	dec
11	undec	12	dodec	13	tridec
14	tetradec	15	pentadec	16	hexadec
17	heptadec	18	octadec	19	nonadec
20	eicos	21	heneicos	22	docos
23	tricos	24	tetracos	25	pentacos
26	hexacos	27	heptacos	28	octacos
29	nonacos	30	triacont	31	hentriacont
32	dotriacont	33	tritriacont	34	tetratriacont
35	pentatriacont	36	hexatriacont	37	heptatriacont
38	octatriacont	39	nonatriacont	40	tetracont
41	hentriacont	42	dotetracont	43	tritetracont
44	tetratetracont	45	pentatetracont	46	hexatetracont
47	heptatetracont	48	octatetracont	49	nonatetracont
50	pentacont				

Figure S1. Available template structures for glycerophospholipids (GP). Template IDs containing head group specification are shown below the structures.

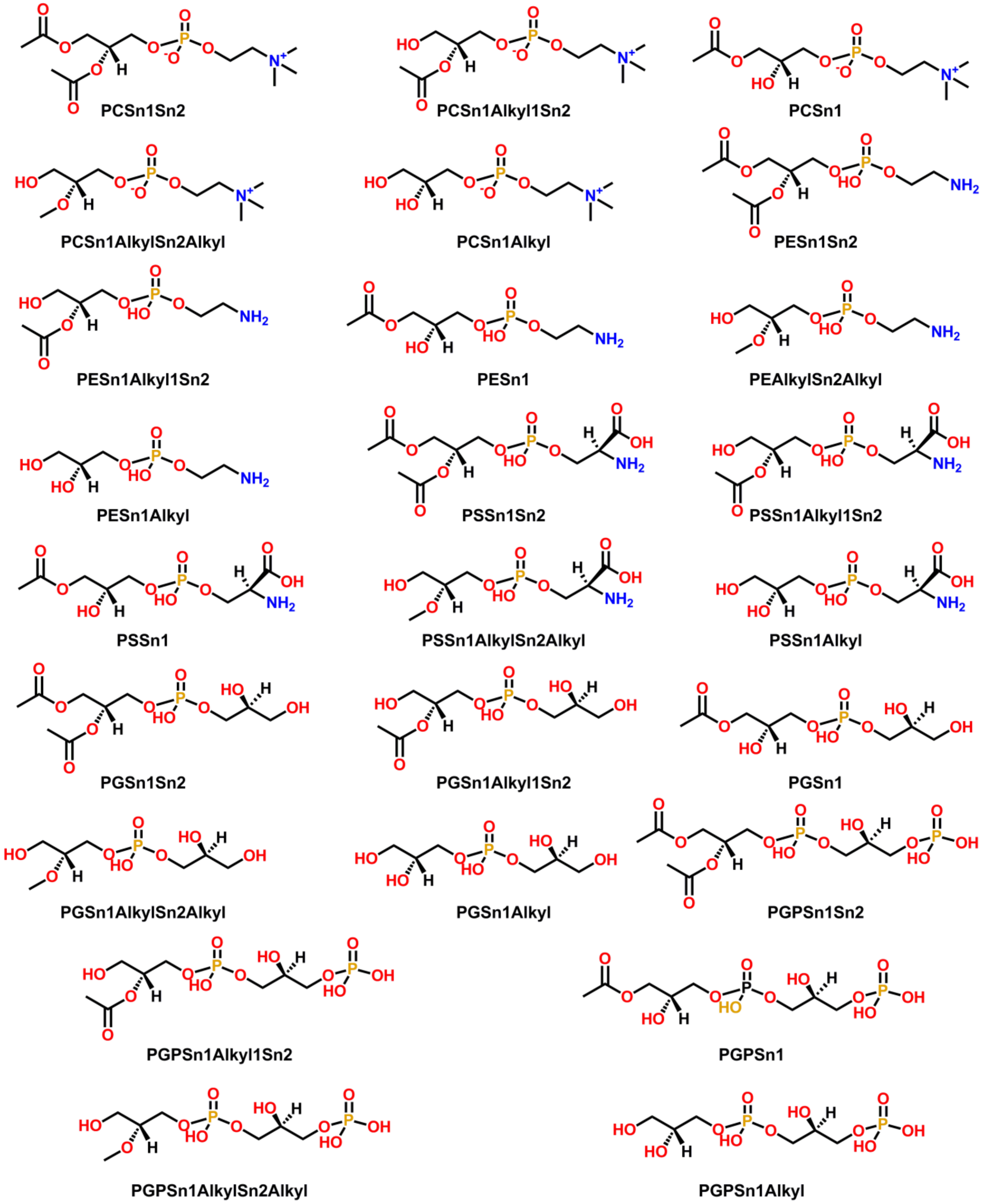

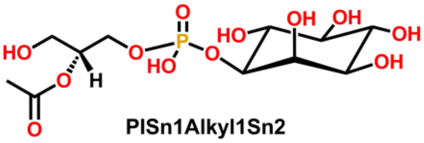

PISn1Alkyl1Sn2

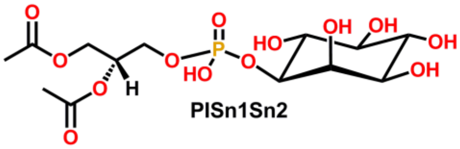

PISn1Sn2

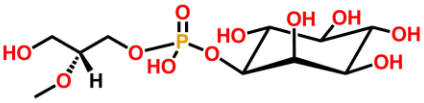

PISn1AlkylSn2Alkyl

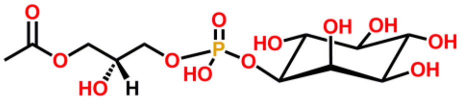

PISn1

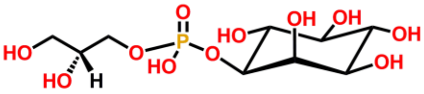

PISn1Alkyl

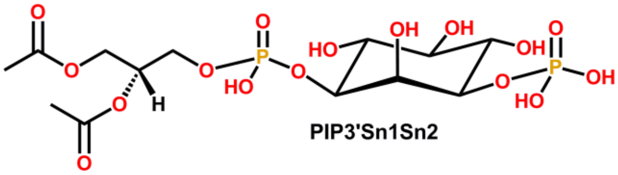

PIP3'Sn1Sn2

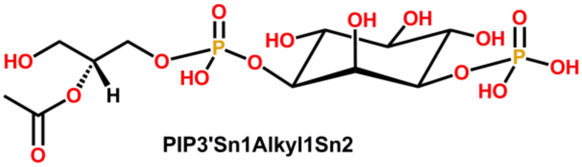

PIP3'Sn1Alkyl1Sn2

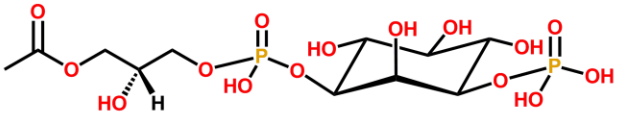

PIP3'Sn1

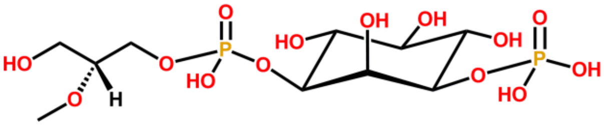

PIP3'Sn1AlkylSn2Alkyl

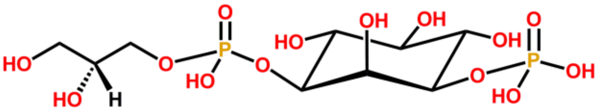

PIP3'Sn1Alkyl

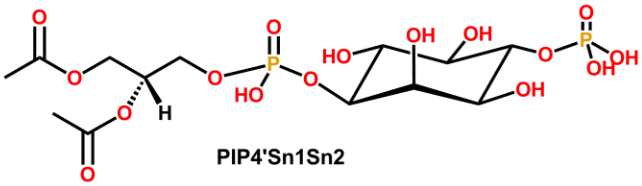

PIP4'Sn1Sn2

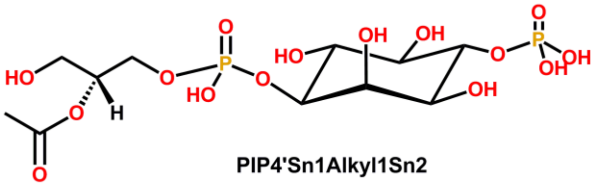

PIP4'Sn1Alkyl1Sn2

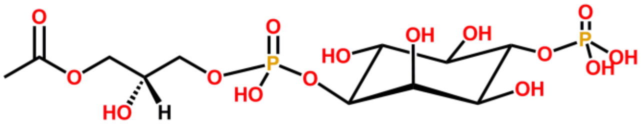

PIP'4Sn1

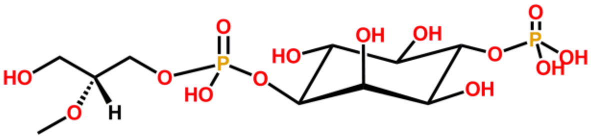

PIP4'Sn1AlkylSn2Alkyl

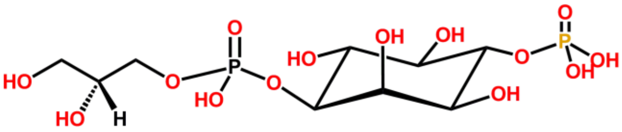

GPIP4'Sn1Alkyl

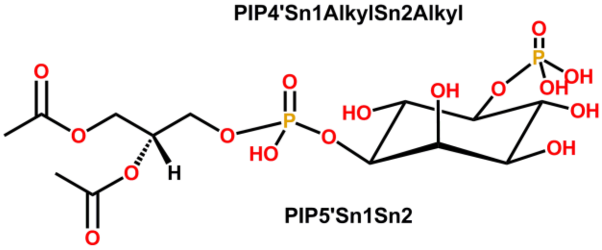

PIP5'Sn1Sn2

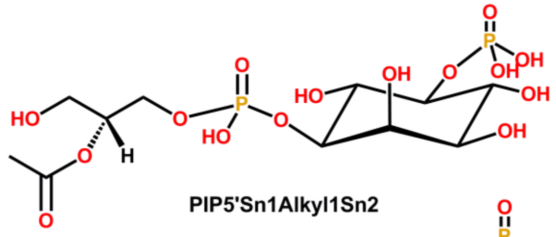

PIP5'Sn1Alkyl1Sn2

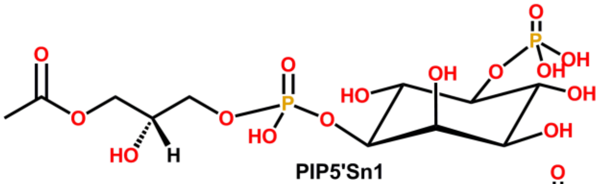

PIP5'Sn1

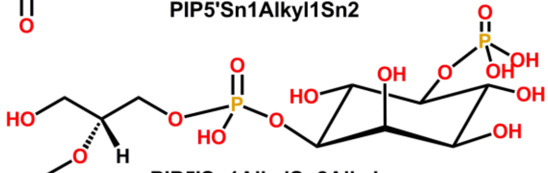

PIP5'Sn1AlkylSn2Alkyl

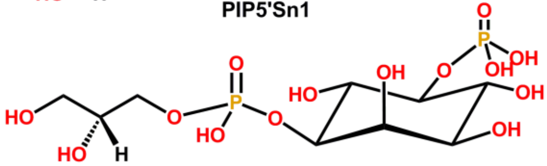

PIP5'Sn1Alkyl

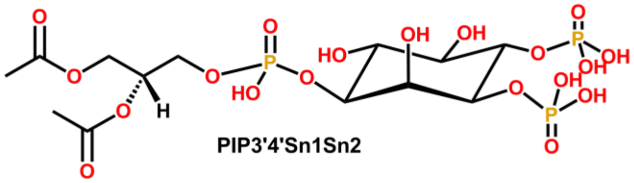

PIP3'4'Sn1Sn2

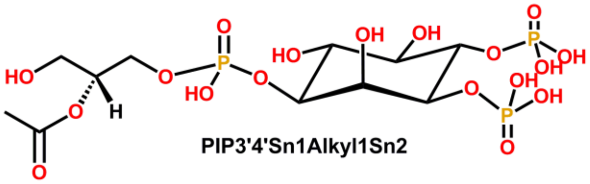

PIP3'4'Sn1Alkyl1Sn2

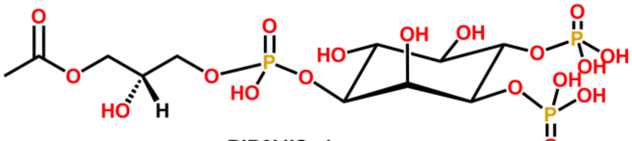

PIP3'4'Sn1

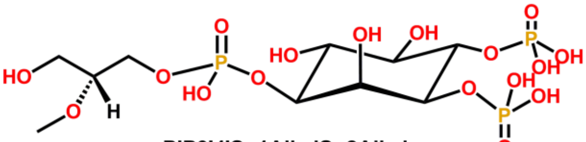

PIP3'4'Sn1AlkylSn2Alkyl

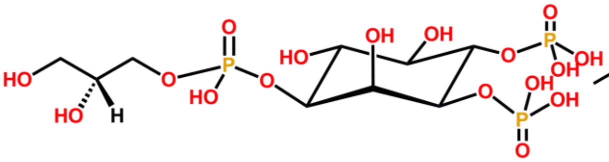

PIP3'4'Sn1Alkyl

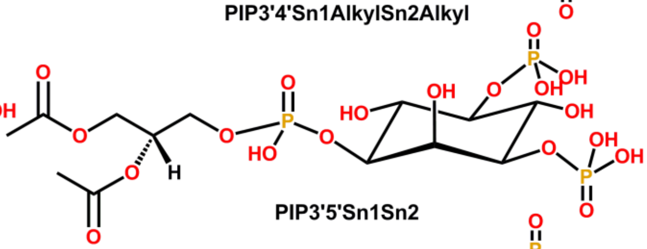

PIP3'5'Sn1Sn2

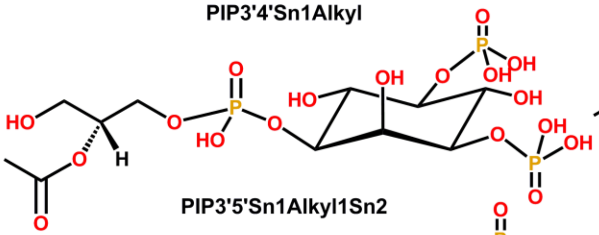

PIP3'5'Sn1Alkyl1Sn2

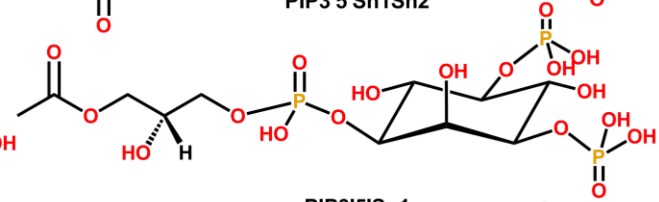

PIP3'5'Sn1

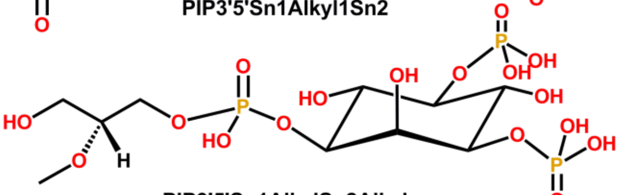

PIP3'5'Sn1AlkylSn2Alkyl

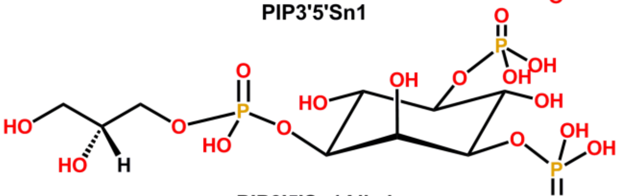

PIP3'5'Sn1Alkyl

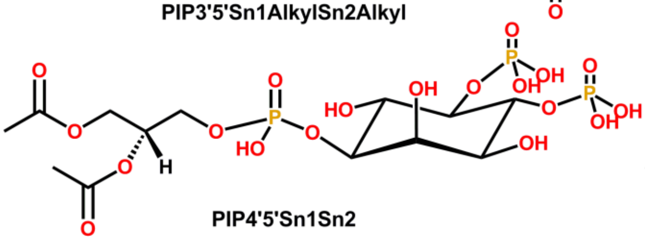

PIP4'5'Sn1Sn2

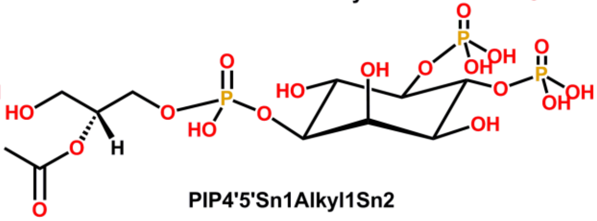

PIP4'5'Sn1Alkyl1Sn2

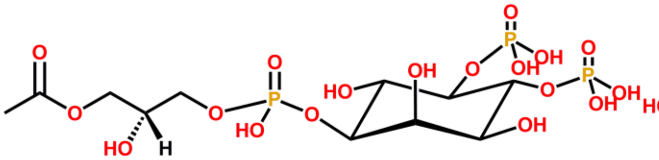

PIP4'5'Sn1

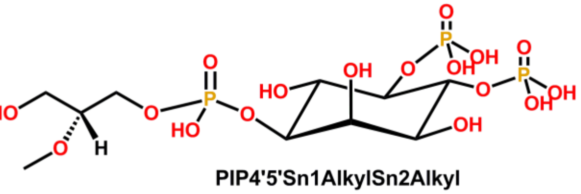

PIP4'5'Sn1AlkylSn2Alkyl

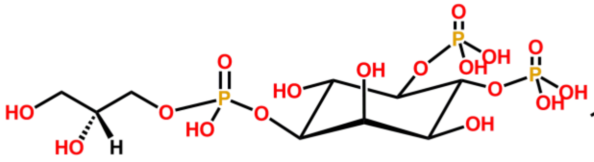

PIP4'5'Sn1Alkyl

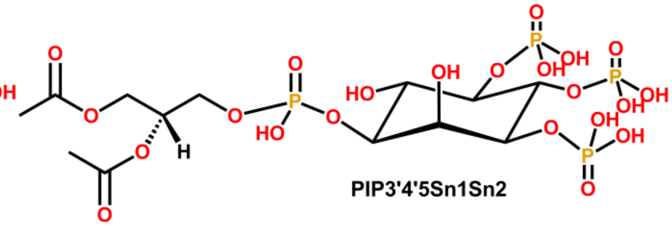

PIP3'4'5Sn1Sn2

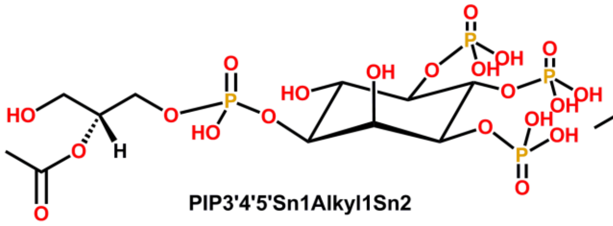

PIP3'4'5'Sn1Alkyl1Sn2

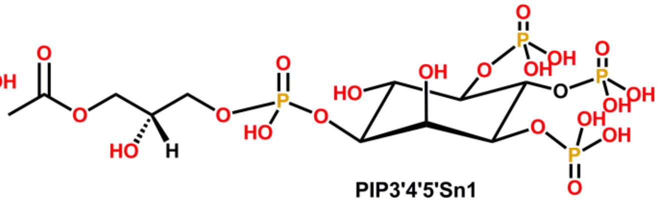

PIP3'4'5'Sn1

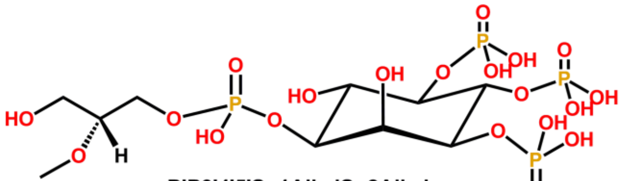

PIP3'4'5'Sn1AlkylSn2Alkyl

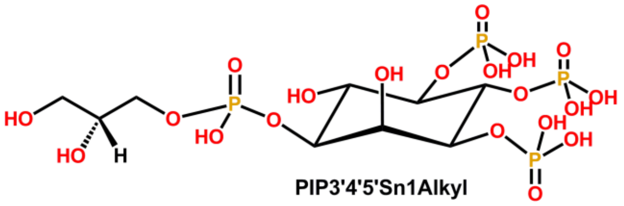

PIP3'4'5'Sn1Alkyl

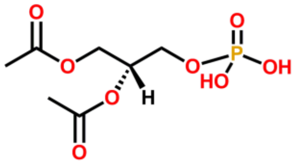

PASn1Sn2

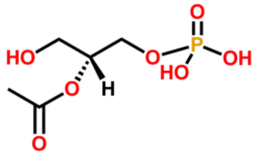

PASn1Alkyl1Sn2

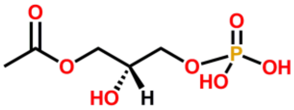

PASn1

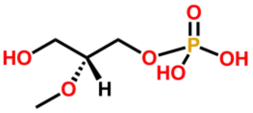

PASn1AlkylSn2Alkyl

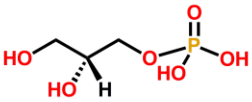

PASn1Alkyl

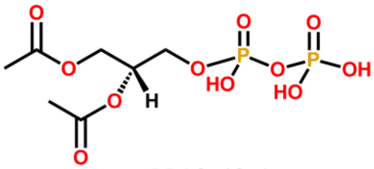

PPASn1Sn2

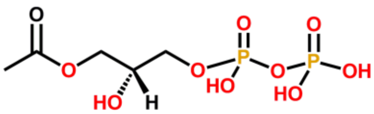

PPASn1

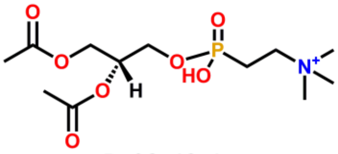

PnCSn1Sn2

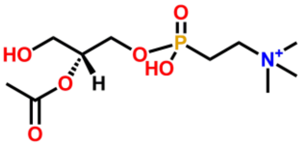

PnCSn1Alkyl1Sn2

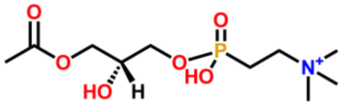

PnCSn1

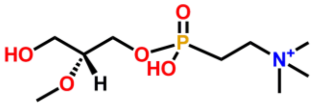

PnCSn1AlkylSn2Alkyl

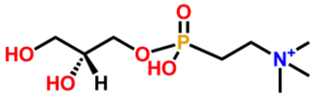

PnCSn1Alkyl

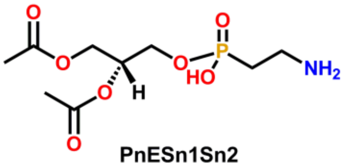

PnESn1Sn2

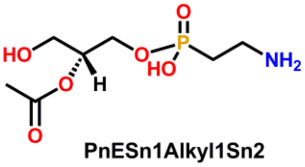

PnESn1Alkyl1Sn2

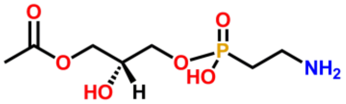

PnESn1

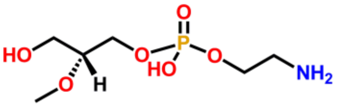

PnESn1AlkylSn2Alkyl

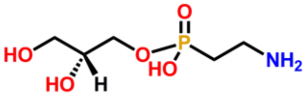

PnESn1Alkyl

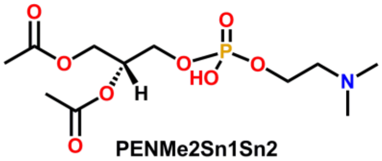

PENMe2Sn1Sn2

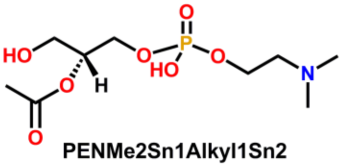

PENMe2Sn1Alkyl1Sn2

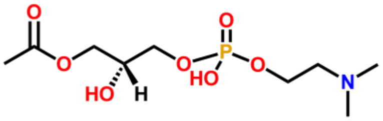

PENMe2Sn1

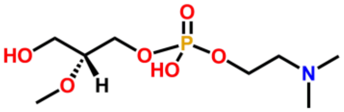

PENMe2Sn1AlkylSn2Alkyl

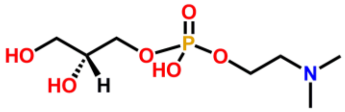

PENMe2Sn1Alkyl

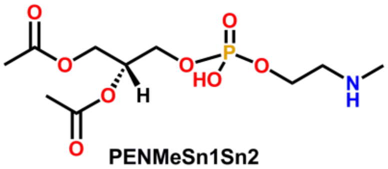

PENMeSn1Sn2

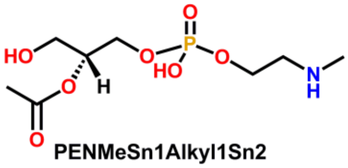

PENMeSn1Alkyl1Sn2

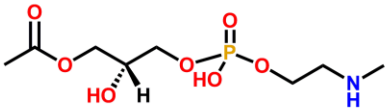

PENMeSn1

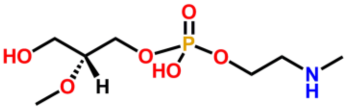

PENMeSn1AlkylSn2Alkyl

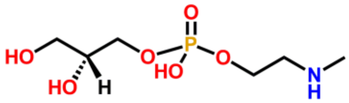

PENMeSn1Alkyl

Figure S2. Available template structures for sphingolipids (SP). Template IDs containing head group specification are shown below the structures. A representative example of an abbreviation matching a template is shown below the template structure.

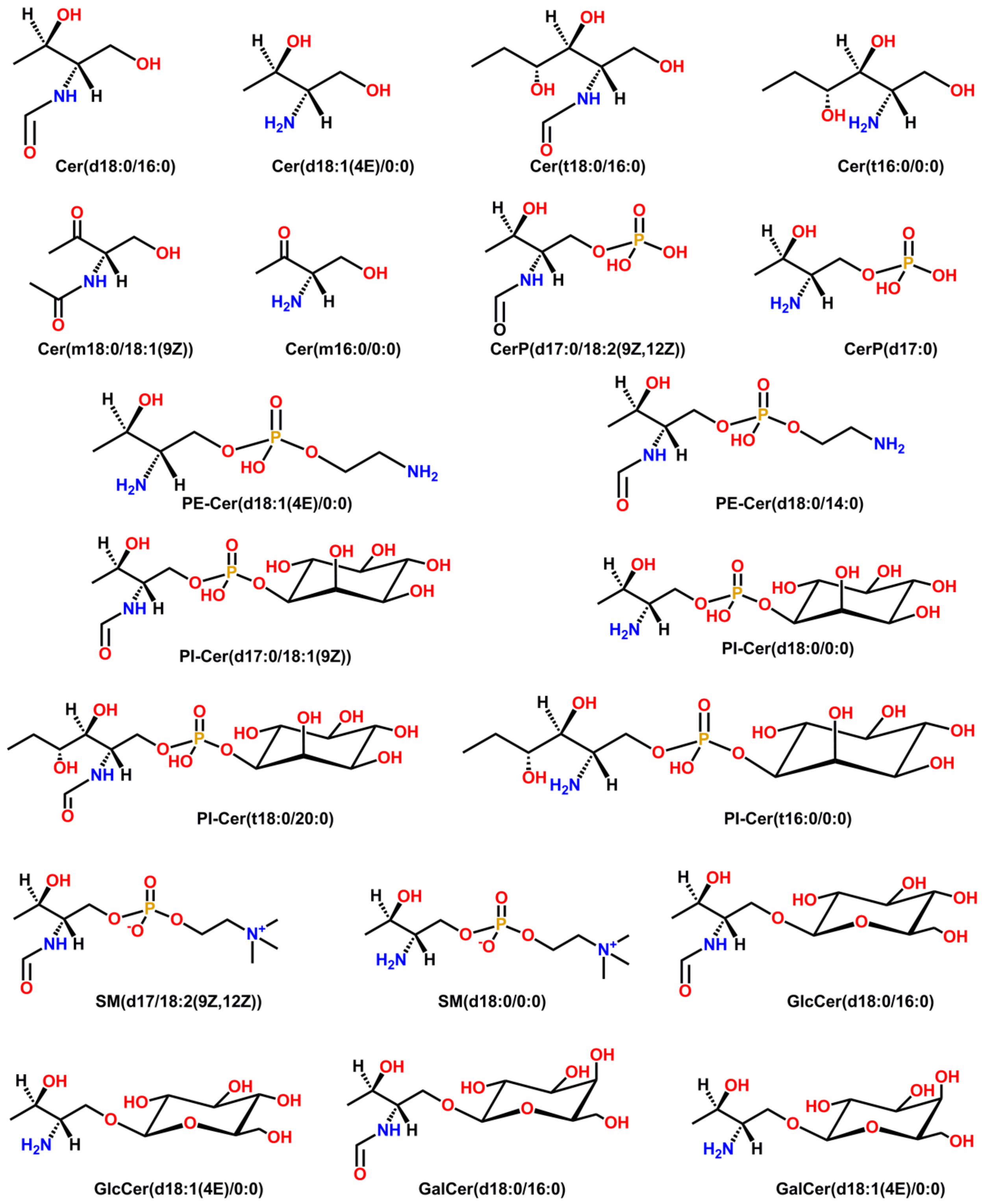

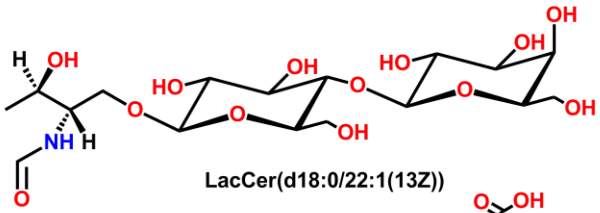

LacCer(d18:0/22:1(13Z))

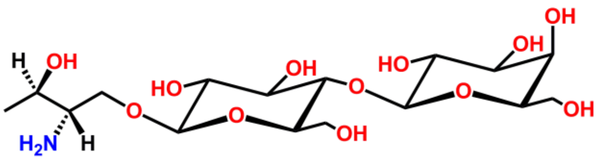

LacCer(d18:1(4E)/0:0)

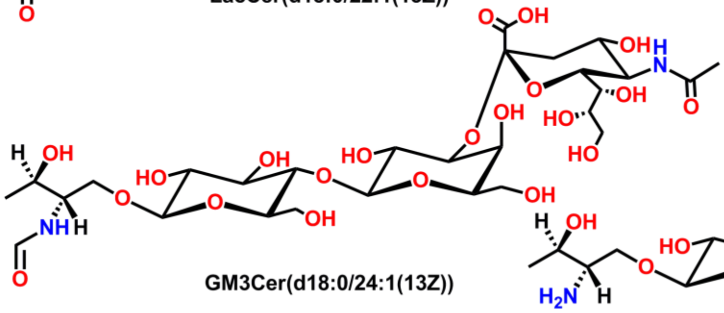

GM3Cer(d18:0/24:1(13Z))

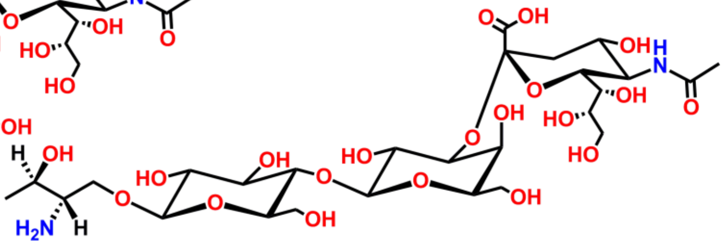

GM3Cer(d18:1(4E)/0:0)

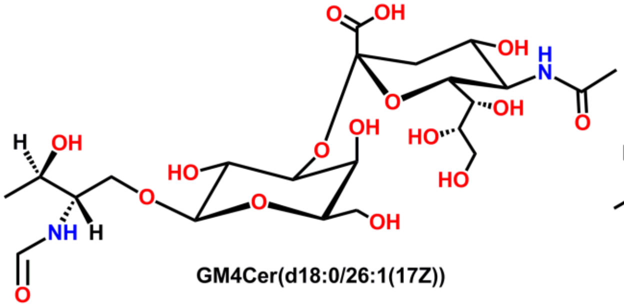

GM4Cer(d18:0/26:1(17Z))

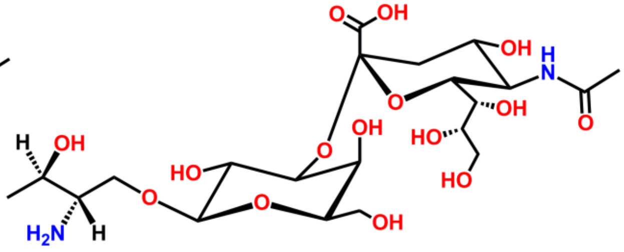

GM4Cer(d17/0:0)

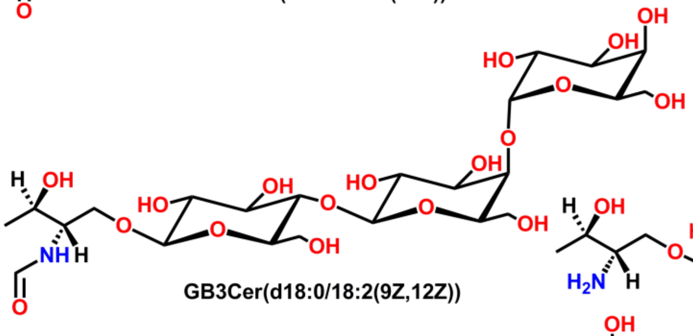

GB3Cer(d18:0/18:2(9Z,12Z))

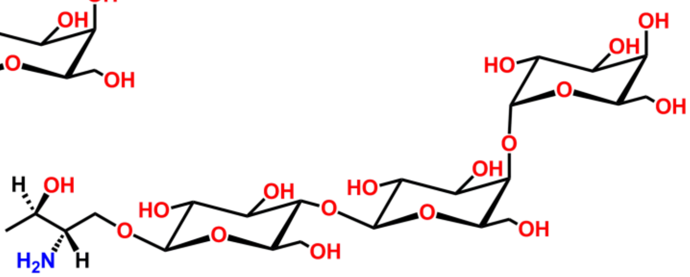

GB3Cer(d16:0/0:0)

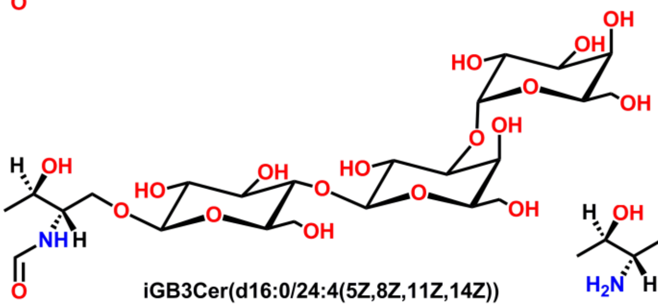

iGB3Cer(d16:0/24:4(5Z,8Z,11Z,14Z))

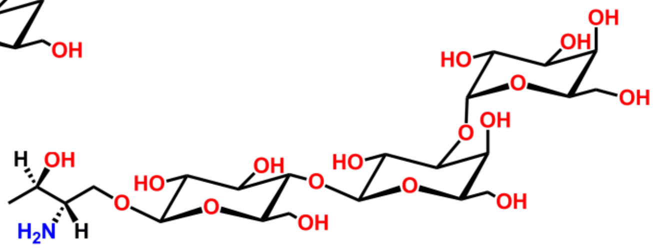

iGB3Cer(d18:1(4E)/0:0)

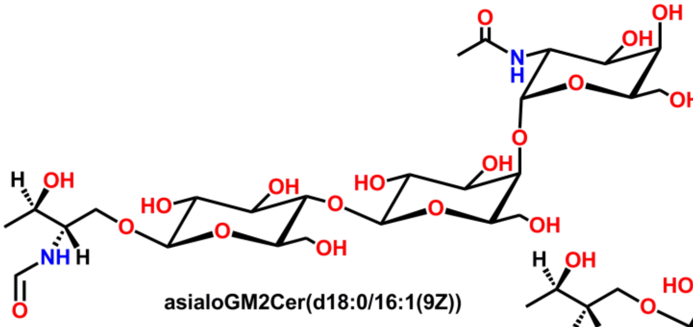

asialoGM2Cer(d18:0/16:1(9Z))

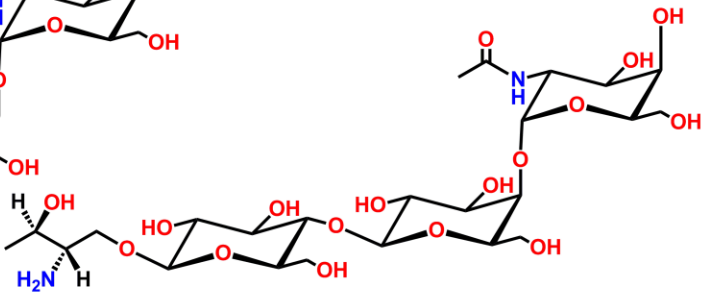

asialoGM2Cer(d18:1(4E)/0:0)

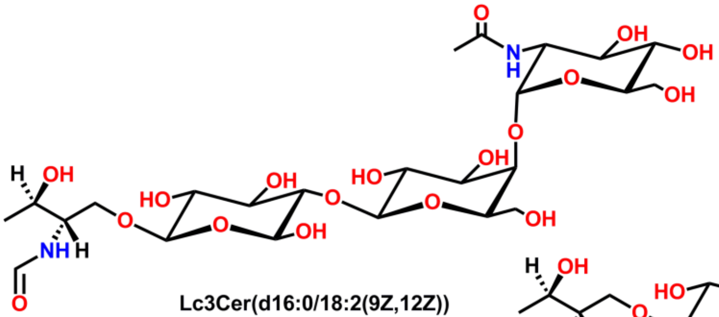

Lc3Cer(d16:0/18:2(9Z,12Z))

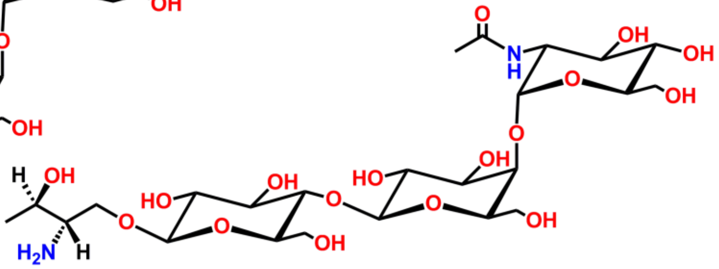

Lc3Cer(d17:0/0:0)

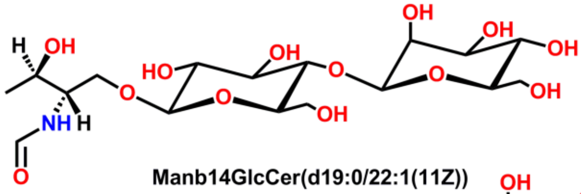

Manb14GlcCer(d19:0/22:1(11Z))

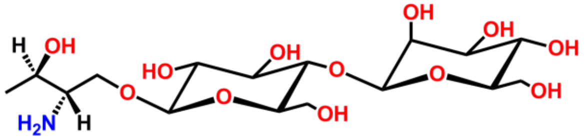

Manb14GlcCer(d19:0/0:0)

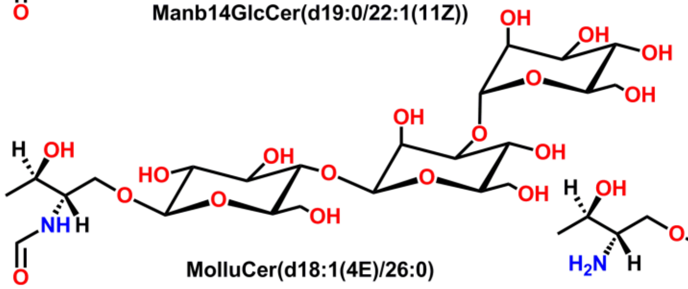

MolluCer(d18:1(4E)/26:0)

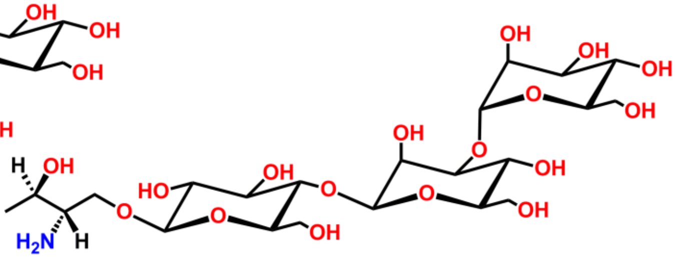

MolluCer(d18:0/0:0)

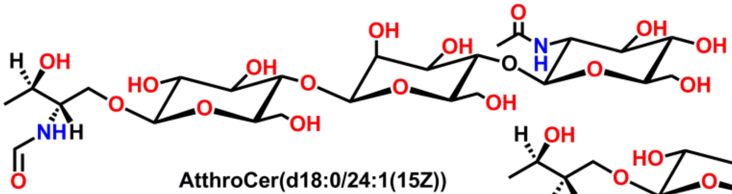

AtthroCer(d18:0/24:1(15Z))

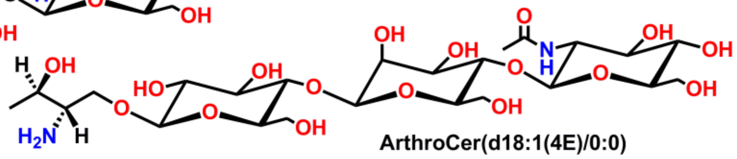

ArthroCer(d18:1(4E)/0:0)
